# A posteriori dietary patterns in 71-year-old Swedish men and the prevalence of sarcopenia 16 years later

**DOI:** 10.1017/S0007114521003901

**Published:** 2022-09-14

**Authors:** Mikael Karlsson, Wulf Becker, Tommy E. Cederholm, Liisa Byberg

**Affiliations:** 1 Department of Public Health and Caring Sciences, Clinical Nutrition and Metabolism, Uppsala University, Uppsala, Sweden; 2 Department of Surgical Sciences, Medical Epidemiology, Uppsala University, Uppsala, Sweden

**Keywords:** Sarcopenia, Dietary pattern, Principal component analysis, Cohort, Longitudinal, Muscle mass

## Abstract

The role of diet in sarcopenia is unclear, and results from studies using dietary patterns (DP) are inconsistent. We assessed how adherences to a posteriori DP are associated with the prevalence of sarcopenia and its components 16 years later. Four DP were defined in the Uppsala Longitudinal Study of Adult Men at baseline (*n* 1133, average age 71 years). Among 257 men with information at follow-up, 19 % (*n* 50) had sarcopenia according to the European Working Group on sarcopenia in Older People 2 definition. Adherence to DP2 (mainly characterised by high intake of vegetables, green salad, fruit, poultry, rice and pasta) was non-linearly associated with sarcopenia; adjusted OR and 95 % CI for medium and high *v*. low adherence: 0·41 (0·17, 0·98) and 0·40 (0·17, 0·94). The OR per standard deviation (sd) higher adherence to DP2 was 0·70 (0·48, 1·03). Adjusted OR (95 % CI) for 1 sd higher adherence to DP1 (mainly characterised by high consumption of milk and cereals), DP3 (mainly characterised by high consumption of bread, cheese, marmalade, jam and sugar) and DP4 (mainly characterised by high consumption of potatoes, meat and egg and low consumption of fermented milk) were 1·04 (0·74, 1·46), 1·19 (0·71, 2·00) and 1·08 (0·77, 1·53), respectively. There were no clear associations between adherence to the DP and muscle strength, muscle mass, physical performance or sarcopenia using EWGSOP1 (sarcopenia *n* 54). Our results indicate that diet may be a potentially modifiable risk factor for sarcopenia in old Swedish men.

Sarcopenia is recognised as a significant health concern in older individuals^(1)^, including physical impairment, risk of falls and fractures, disability^(2)^, reduced quality of life^(3,4)^ and higher in-hospital and 1-year mortality^(2)^. Sarcopenia was initially defined as an age-related decline in muscle mass. Later, muscle function has been included in most sarcopenia definitions^(5–9)^, since muscle strength, muscle power and physical performance are observed as stronger predictors of clinically relevant outcomes, e.g. functional status, falls and mortality^(10–13)^. Even if it may arise in mid-life, it arises generally as an age-related progression^(14)^.

Since sarcopenia influences the individual’s health and well-being, it is of great importance to identify contributing modifiable causes. Most studies have focused on the components in the definition as an outcome, rather than sarcopenia itself. Increased physical activity has been reported to have positive effects on muscle mass and improves overall physical function^(15,16)^. However, the effect of diet is less clear, and data are mostly limited to cross-sectional studies^(17,18)^. Studies examining the role of diet in sarcopenia have predominantly focused on single nutrients or food items^(18)^. However, this might not be the best approach when examining effects on chronic diseases in the non-deficient state^(19,20)^. From a public health perspective, it is difficult to provide dietary advice based solely on nutrients, as the nutrient intake is associated with dietary patterns (DP)^(21)^. It is therefore difficult to examine effects of single nutrients or food items without potential influences of both foods and DP^(22)^. Cross-sectional and longitudinal studies suggest that so-called healthy DP contribute to maintain physical performance in ageing. However, there is limited evidence concerning the relation to muscle mass and muscle strength^(17)^.

In general, a higher adherence to predefined ‘healthy DP’ have been associated with lower prevalence of sarcopenia, reported both by us^(23)^ and others^(24–26)^, although some ‘healthy DP’ were not associated with sarcopenia in the same study populations^(23,24)^. Established predefined dietary indexes are often based on current knowledge of the association between diet and the outcome. As the knowledge of associations between diet and sarcopenia is limited, a more exploratory technique, such as data driven so-called a posteriori approaches, might be advantageous. Therefore, we assessed the associations between adherences to a posteriori DP, among 71-year-old Swedish men, and the prevalence of sarcopenia, and its components, 16 years later.

## Materials and methods

### Ethical Approval

This study was conducted according to the guidelines laid down in the Declaration of Helsinki, and all procedures involving human subjects were approved by the Regional Ethical Review Board at Uppsala University; Dnr 251/90 & 2007/338. Written informed consent was obtained from all subjects.

### Study population

This study is based on the Uppsala Longitudinal Study of Adult Men (ULSAM)^(27)^, an ongoing population-based cohort study that started in 1970 when all men born 1920–1924 and living in Uppsala County were invited to a health examination; 2322 men participated (participation rate 82 %), outlined in Supplementary Fig. 1. Reexaminations of these men have been carried out at the approximate ages of 60, 70, 77, 82, 88 and 93 years.

Baseline for the present study was the third investigation cycle conducted in 1991–1995 (ULSAM3), at the average age of 71 years, when 1221 men participated (participation rate 73 %) in the reinvestigation. In order to define the DP, availability of dietary data at baseline (*n* 1138) and a reported energy intake between 800 and 4000 kcal/d^(28)^ (*n* 1133) were required. Follow-up was the sixth investigation cycle conducted in 2008–2009 (ULSAM6), at the average age of 87, when 354 men participated (participation rate 58 %). Of these 354 men, 39 had missing data on dietary intake in ULSAM3 and were excluded. The examination at ULSAM6 included physical function tests and 290 of the participants completed body composition measurement using whole-body dual-energy X-ray absorptiometry, as described below. In total, 315 men were included in the analyses of DP and the constituent variables in the definition of sarcopenia. In 257 men, data were available to define sarcopenia according to the latest EWGSOP definition^(29)^, and none of these participants had missing values in covariates.

### Dietary assessment, food grouping and a posteriori dietary pattern

Information on dietary intake was collected during seven consecutive days using a pre-coded menu book (online Supplementary Material 1) from the Swedish National Food Agency, giving preprinted alternatives for food items, dishes and when the meals are consumed. Food intake was reported in household measures or in predefined portion sizes. Consumptions not pre-coded in the menu book were to be reported in free-text^(30)^. A dietician or a trained nurse instructed the participant on how to use the menu book, which also included written instructions and pictures of portions. Dietary data were analysed with commercial software using a food composition database from the Swedish National Food Agency (SLV version 1990). The menu book was validated in a subgroup of the study population^(31)^, by comparison with open-ended weighed food records, displaying a larger proportion of participants under-reporting energy intake compared to weighed food records, but with moderate to high agreements between the methods regarding intake of macro and micro-nutrients.

DP were defined based on the 1133 men (ULSAM3) having a reported dietary intake during 7 d. Mean intake of the seven recoding days of reported food items was categorised into twenty-six food groups, based on the food item’s nutrient composition and how the food item is used in cooking or eaten in the population studied (online Supplementary Table 1). For example, milk and fermented milk products were divided into different groups because fermented milk is commonly used together with cereals at breakfast or as a light meal, and milk has been used as a beverage in Sweden since the 1930s^(32,33)^. Cold meats include food items often used on bread at breakfast or as light meals. In Sweden, potatoes are used in a similar way to rice and pasta in other countries and are therefore excluded from the food group Vegetables. Tea, coffee and tap water were excluded as they did not significantly contribute neither to energy intake nor to DP separation. Another reason for excluding tap water was due to probable misreporting. Cream (385 men had a reported intake, mean intake < 6 g/d) and vegetable oils (three men had a reported intake, mean intake < 5 g/d) were excluded due to infrequent consumption. Butter and margarine were excluded as they may be misclassified (in Sweden margarine is often called ‘butter’ in everyday language), and fat used for cooking was included in dishes. The exclusion of these food items rendered a greater proportion of variance explained and more distinct DP, but did not affect the characteristic features of the DP. In order to preserve a wider variation in dietary intake, the dietary intake was not adjusted for the total energy intake when DP were defined^(34)^. However, total energy intake was included in the multivariable adjusted analyses of DP in relation to the outcomes.

DP were defined using principal component analysis, which reduces larger amounts of observed variables to a smaller number of principal components while maximising the variance and identifying structures in the observed data. Bartlett’s test of sphericity (< 0·001), indicating that the variables are related, and Kaiser-Meyer-Olkin measure of sampling (0·557), indicating the proportion of variances that might be caused by underlying factors, together indicated that the factor analysis was suitable for structure detection and useful with our data. We applied a commonly used threshold^(34,35)^ (principal components loadings > 0·30 and < –0·30 for each food group) for interpretation of the DP (online Supplementary Table 2); however, DP were not labelled on the basis of this interpretation.

The top four principal components were selected based on a combined assessment of a break point (elbow) in the scree plot, eigenvalues (> 1·5) and domestic-cultural knowledge. The identified principal components were rotated with varimax, creating orthogonal (uncorrelated) factors that form the defined DP for this study. The four chosen DP (DP1, DP2, DP3 and DP4) accounted for 28 % of the total variance.

Using the postestimation command *predict*, each participant was given a factor score, based on the retained factors, to reflect the agreement with each DP. Hence, a high factor score indicates a high intake from food groups that loaded positively and low intake of food groups loading negatively on the given DP. Based on the factor scores’ tertile percentiles in the total population of 1133 men at baseline, participants were categorised as having low, medium or high adherence to a given DP.

### Definition of sarcopenia

The European Working Group on Sarcopenia in Older People (EWGSOP) proposed diagnostic criteria for sarcopenia in 2010^(36)^, denoted as EWGSOP1. These criteria were revised in 2019^(29)^ and denoted as EWGSOP2. Sarcopenia defined according to EWGSOP2 was the main outcome of this study and defined as the combination of low muscle strength (reduced hand grip strength, < 27 kg^(37)^ and/or five-times chair stand test, > 15 s)^(38)^ and low muscle mass (appendicular lean mass index, lean mass index, < 7·0 kg/m^2^)^(39)^. Severity grading of sarcopenia was not done.

Sarcopenia defined according to EWGSOP1 was used in sensitivity analysis, to allow comparison with previous published studies. Participants were categorised as having sarcopenia, i.e. having low muscle mass together with either low hand grip strength and/or low gait speed or otherwise as not having sarcopenia. The chosen cut-off values were for appendicular lean mass index < 7·26 kg/m^2^ (low muscle mass)^(40)^, hand grip strength < 30 kg (low muscle strength)^(41)^ and gait speed < 0·8 m/s (low physical performance)^(41)^.

### Muscle strength, muscle power and physical performance

Handgrip strength was measured using an adjustable hydraulic hand dynamometer (Fabrication Enterprises, White Plains), considered to measure grip strength with the same precision as the Jamar hydraulic hand dynamometer^(42)^. Participants were sitting on a chair with the arm supported, shoulder relaxed, elbow at an angle of 90°, the wrist in a neutral position and feet on the floor. Measurement started with the dominant hand and the highest of three results for each hand was recorded, whereof the highest value, regardless if dominant hand or not, was used. In total, 301 participants performed the test at follow-up; two participants were unable to perform the test due to pain or disease and 12 due to unknown reasons.

The chair stand test included five chair rises, as fast as possible in a safe manner, with arms crossed over the chest. The duration was measured, to the nearest 0·5 s, from the first rise until seated ageing after the fifth rise. A total of 241 participants performed five rises, sixty-six participants tried but were unable to perform the test, four participants declined to perform the test or were unable due to medical reasons and four participants had missing value due to unknown reasons. Participants who failed the chair stand test were handled as having a result above the cut-off (> 15 s) in the sarcopenia definition but were excluded in analyses using chair stand test as outcome.

A self-chosen comfortable walking speed was measured using the intermediate 6 m of a distance of 10 m with no obstacles. Participants were allowed to use walking aid if they preferred to. In total, 257 participants performed the test at follow-up. Reasons for missing data included home visit and therefore not tested (*n* 50), unable to perform the test due to disease or lack of strength (*n* 7) or due to unknown reason (*n* 1). The fifty-eight participants with missing data were excluded from analyses using gait speed as outcome.

### Anthropometry and body composition measurements

Body height was measured to the nearest centimeter and body weight to the nearest 0·1 kg. BMI was calculated as the ratio of the weight to the squared height (kg/m^2^).

Lean muscle mass was measured using dual-energy X-ray absorptiometry (DPX Prodigy, Lunar corp.). Precision errors of dual-energy X-ray absorptiometry measurements in the present laboratory have been calculated to be 1·5 % for total fat mass and 1·0 % for total lean mass^(43)^. Appendicular lean mass index was calculated as the ratio of appendicular muscle mass (lean mass of legs and arms) to the squared height (kg/m^2^)^(40)^.

### Covariates used for regression analyses

Questionnaires on education, physical activity and smoking habits were completed at baseline (ULSAM3) under standardised conditions, as earlier described^(44)^. Information on educational level was categorised by years in school (6–7 years, 8–13 years or > 13 years). The leisure-time physical activity was defined as sedentary, moderate, regular or athletic^(45)^ using a validated questionnaire^(46)^. Physical activity was then categorised in three subgroups: low (sedentary and moderate), medium (regular) or high (athletic). Smoking status was categorised as never, former or current smoker.

The follow-up period was defined as the period from the date of examination at baseline to the date of dual-energy X-ray absorptiometry measurement at follow-up.

Charlson’s unweighted Comorbidity Index^(47,48)^ was calculated based on in-patient diagnoses from patient records recorded before baseline. The comorbidity score was then dichotomised according to score (0 and ≥ 1).

Age, follow-up period, reported energy intake and BMI were used as continuous variables in statistical analyses. Education, physical activity, smoking and the Charlson Comorbidity Index were used as categorical variables.

### Statistical analyses

The association between each DP and the prevalence of sarcopenia was analysed using logistic regression models estimating OR and their 95 % CI.

Further, we assessed the associations between DP and the four components in the definition of sarcopenia (i.e. handgrip strength, chair stand test, appendicular lean mass index and gait speed) using linear regression analyses estimating beta coefficients and their 95 % CI.

For each of these analyses, linear and non-linear associations were explored. In the linear model, each DP was entered as a standardised continuous variable with mean 0 and 1 sd increments. Non-linear associations were first explored with each DP as a categorical variable, according to low, medium or high adherence, based on their factor scores and tertile limits determined in the total baseline population of 1133 men. A joint Wald test was performed to evaluate whether factor indicators are equal to 0. Next, each DP was entered as a standardised continuous variable with 1 sd increments using restricted cubic splines with three knots placed at the 10th, 50th and 90th percentiles of the DP distribution, as recommended by Harrell^(49)^, and with the median used as reference point, illustrating potential nonlinear associations; results are presented as graphs.

A directed acyclic graph approach was applied using DAGitty^(50)^ (online Supplementary Fig. 2) to identify potential confounders to be included in the multivariable models^(51)^. The directed acyclic graph is a graphical presentation of the assumed causal relationships between factors relevant for the current research question. Based on these assumptions and assuming no residual confounding, DAGitty provides information about minimal sufficient adjustment sets for estimating the total effect of an exposure on the outcome. We applied three models. Model 1 was unadjusted for potential confounders. Model 2 was adjusted for age at baseline, follow-up period, reported energy intake at baseline, education, physical activity level at baseline, smoking and morbidity at baseline. Model 3 was further adjusted for BMI at baseline. Model 3 is in agreement with the minimal sufficient adjustment set suggested by DAGitty.

All analyses were performed in Stata version 15.1 (Stata Corp).

## Results

### Characteristics of the studied population

There were some differences in baseline characteristics between the main study population of 257 men with follow-up information and the total group of 1133 men with dietary information at baseline (online Supplementary Table 3). The group of 257 men had a lower BMI, a higher reported energy intake, a smaller proportion were smokers and had more years of education, higher reported physical activity level and a lower Charlson Comorbidity Index. Differences in average dietary intake were small and not clinically relevant (Table 1 and online Supplementary Table 4).

**Table 1.** Dietary intake among men included in the main analysis, displayed as food groups at baseline by adherence to each dietary pattern at baseline (Mean values and standard deviations).

			DP 1						DP 2						DP 3						DP 4					
	Total		Low		Medium		High		Low		Medium		High		Low		Medium		High		Low		Medium		High	
	Mean	sd	Mean	sd	Mean	sd	Mean	sd	Mean	sd	Mean	sd	Mean	sd	Mean	sd	Mean	sd	Mean	sd	Mean	sd	Mean	sd	Mean	sd
Food groups*	*n* 257		*n* 98		*n* 68		*n* 91		*n* 74		*n* 91		*n* 92		*n* 67		*n* 81		*n* 109		*n* 100		*n* 83		*n* 74	
Cheese	35	21	35	20	40	24	32	20	32	20	33	20	40	22	21†	14†	29†	15†	48†	21†	34	18	35	23	36	23
Bread	108	41	107	34	115	48	104	43	101	39	104	36	119	46	73†	19†	101†	33†	136†	38†	99	32	107	43	122	47
Fermented milk	99	102	131	102	108	108	58	81	83	98	98	103	113	103	59	71	93	92	129	114	144†	108†	82†	84†	59†	87†
Milk	230	188	114†	110†	206†	144†	373†	191†	232	185	226	198	233	182	204	175	242	194	237	192	200	164	235	182	265	219
Soup	28	40	27	40	30	40	26	40	23	31	22	30	37	51	25	47	24	36	32	37	35	44	22	31	24	41
Pancake	21	26	11	21	19	19	33	30	27	30	16	21	21	25	15	21	22	23	25	29	26	26	20	27	15	23
Quiche and pie	8	17	8	18	11	20	6	15	11	19	8	18	6	15	5	12	6	15	12	21	12	21	8	17	4	12
Salad with protein	7	15	14	21	3	8	1	3	2	5	5	13	12	20	6	15	6	16	7	15	7	14	6	15	7	17
Cold meats	14	14	15	13	18	16	10	12	12	12	14	14	15	15	11	11	13	14	16	15	10	10	13	13	20	17
Meat	65	28	61	26	62	27	71	30	66	24	66	26	63	32	63	28	65	27	66	29	50†	23†	62†	20†	88†	27†
Poultry	5	8	7	9	6	9	2	6	1†	2†	4†	6†	10†	11†	6	10	5	8	5	8	5	8	4	7	6	10
Egg	14	17	18	21	13	14	11	14	15	18	14	16	14	18	13	17	13	19	15	16	7†	9†	13†	16†	24†	22†
Seafood	4	8	4	9	4	6	4	8	2	5	5	8	4	8	3	7	4	8	4	7	4	7	3	8	4	8
Vegetables	26	26	23	21	24	23	29	33	13†	11†	19†	13†	42†	36†	25	31	28	28	24	22	26	27	19	19	32	31
Green salad	34	31	42	35	30	25	29	29	18†	18†	32†	26†	49†	36†	25	23	37	34	38	32	32	31	36	32	36	29
Legumes	16	26	8	17	13	22	28	31	19	29	14	22	17	25	15	25	17	26	17	26	13	21	20	29	18	26
Juice	30	58	27	58	39	56	28	59	15	33	17	33	56	80	15	33	29	57	41	67	47	77	19	34	21	43
Fruit	124	101	110	85	121	93	141	119	60†	56†	119†	74†	180†	118†	99	91	126	101	137	104	155	111	110	87	98	90
Pasta and rice	20	26	20	22	21	29	19	29	7†	9†	16†	18†	34†	34†	21	25	19	25	20	29	20	23	14	17	27	36
Potato	145	64	127	50	145	51	163	79	140	54	142	57	150	77	125	59	145	53	156	72	117†	50†	148†	48†	179†	79†
Cereals	85	97	23†	33†	56†	56†	174†	102†	80	88	88	95	86	106	79	100	100	105	78	88	74	83	88	98	97	112
Dessert and pastry	92	70	69	52	102	71	108	80	81	59	100	82	92	63	59	62	87	52	115	78	110	69	93	77	65	53
Marmalade, jam and sugar	25	21	21	18	21	19	32	24	28	25	22	19	25	21	14†	12†	20†	14†	35†	26†	28	25	23	20	23	18
Soft drink	44	94	29	83	45	101	59	99	54	97	41	85	39	101	28	65	45	93	53	109	46	97	40	86	47	100
Alcohol content < 4 %	153	143	236†	167†	117†	97†	91†	94†	171	167	166	143	126	119	116	118	166	160	167	142	125	131	159	138	185	158
Alcohol content > 4 %	33	55	65†	72†	19†	26†	10†	22†	32	58	34	47	34	59	33	45	35	59	33	57	33	44	36	66	31	54

Values are presented as mean daily intake in grams (sd).

* Food groups are defined and exemplified in Supplementary Table 1.

† Food groups with principal components loadings > 0·30 and < –0·30.

Characteristics of the 257 men in our main study population at baseline and at follow-up are displayed by DP adherence in Table 2 and in Supplementary Table 5. According to EWGSOP2 (total *n* 257), 50 (19 %) had confirmed sarcopenia. When using the definition EWGSOP1 (total *n* 255) 54 (21 %) had confirmed sarcopenia. Thirty-three participants were defined as having sarcopenia by both definitions. Compared with those without sarcopenia, participants with confirmed sarcopenia (EWGSOP2) had a lower body weight and BMI both at baseline (73·3 kg *v*. 81·0 kg, 24·1 *v*. 26·3 kg/m^2^) and at follow-up (68·6 kg *v*. 78·1 kg, 23·2 *v*. 26·2 kg/m^2^). There were no mean differences between participants with or without sarcopenia in terms of reported intake of energy (kcal/d) or protein (g/d), years of education, reported physical activity level, prevalence of smoking or comorbidity at baseline.

**Table 2.** Characteristics at baseline (mean age 71) and follow-up (mean age 87) of men included in the main analysis and grouped according to low, respectively, high adherence to each dietary pattern at baseline (Mean values and standard deviations; numbers and percentages).

				DP 1				DP 2				DP 3				DP 4			
		Total (*n* 257)		Low (*n* 98)		High (*n* 91)		Low (*n* 74)		High (*n* 92)		Low (*n* 67)		High (*n* 109)		Low (*n* 100)		High (*n* 74)	
		Mean	sd	Mean	sd	Mean	sd	Mean	sd	Mean	sd	Mean	sd	Mean	sd	Mean	sd	Mean	sd
Age (years)	baseline	70·9	0·6	71·0	0·6	71·0	0·6	71·0	0·6	70·9	0·6	70·9	0·6	71·0	0·6	70·9	0·6	70·9	0·7
Follow-up period (years)		15·7	0·7	15·6	0·7	15·6	0·7	15·7	0·7	15·6	0·7	15·6	0·6	15·7	0·7	15·6	0·7	15·6	0·6
Body weight (kg)	baseline	79·5	9·9	80·6	9·9	77·8	10·9	80·0	9·6	78·9	8·7	80·6	12·5	78·4	8·9	80·0	10·6	79·5	9·4
Body weight (kg)	follow-up	76·2	11·5	76·5	11·0	74·7	13·2	76·0	13·3	76·4	10·1	75·7	13·0	76·0	10·1	76·9	12·5	76·1	10·6
BMI (kg/m^2^)	baseline	25·9	2·9	26·0	2·9	25·4	3·0	26·1	3·0	25·6	2·6	26·2	3·5	25·4	2·3	25·9	3·0	25·9	2·8
BMI (kg/m^2^)	follow-up	25·6	3·5	25·5	3·3	25·2	4·0	25·7	4·4	25·5	3·1	25·4	3·9	25·3	2·9	25·7	3·7	25·6	3·4
Appendicular LMI (kg/m^2^)	follow-up	7·5	0·8	7·4	0·8	7·5	0·8	7·4	0·9	7·5	0·6	7·4	0·8	7·5	0·7	7·5	0·8	7·4	0·7
Reported energy intake (kcal/d)	baseline	1853	441	1700	387	2018	459	1744	423	1972	456	1407	248	2152	376	1813	407	1959	470
Reported protein intake (g/d)	baseline	69	16	63	13	74	17	64	14	74	18	55	10	79	15	65	14	76	18
Reported protein intake (g/kg body weight)	baseline	0·88	0·24	0·80	0·20	0·97	0·27	0·81	0·20	0·95	0·25	0·70	0·18	1·02	0·23	0·83	0·19	0·98	0·27
		*n*	%	*n*	%	*n*	%	*n*	%	*n*	%	*n*	%	*n*	%	*n*	%	*n*	%
Years of education (*n*)																			
6–7 years		122	47	29	30	56	62	37	50	36	39	37	55	45	41	39	39	44	59
8–13 years		81	32	34	35	26	29	24	32	30	33	16	24	40	37	33	33	21	28
> 13 years		54	21	35	36	9	10	13	18	26	28	14	21	24	22	28	28	9	12
Physical activity level (*n*)	baseline																		
Low		81	32	34	35	30	33	20	27	28	30	27	40	33	30	32	32	19	26
Medium		156	61	54	55	58	64	51	69	56	61	37	55	67	61	57	57	52	70
High		20	8	10	10	3	3	3	4	8	9	3	4	9	8	11	11	3	4
Smoking (*n*)	baseline																		
Never		104	40	41	42	38	42	29	39	38	41	15	22	49	54	43	43	26	35
Current		28	11	11	11	11	12	8	11	6	7	17	25	5	5	11	11	8	11
Former		125	49	46	47	42	46	37	50	48	52	35	52	55	50	46	46	40	54
Charlson Comorbidity Index (*n*)	baseline																		
0		202	79	77	79	73	80	60	81	72	78	52	78	86	79	77	77	64	86
≥ 1		55	21	21	21	18	20	14	19	20	22	15	22	23	21	23	23	10	14

Values are presented as mean (standard deviation) for continuous measures and number (percentage) for categorical measures.

LMI, lean mass index.

### Dietary patterns

Factor loadings of food groups in the DP (online Supplementary Table 2) correspond to differences in the mean dietary intake, displayed in Supplementary Table 4. The four DP explained 28 % of the total variance. DP1 (7·9 % of total variance) was mainly characterised by high positive loadings for milk and cereals, indicating that participants with high adherence to DP1 consumed greater amounts of these foods, and high negative loadings of alcohol. Thus, participants with high adherence to DP1 consumed, on average, more than three times as much milk, but three times less alcohol as compared with those with low adherence. DP2 (6·9 % of total variance) mainly represents a DP with high consumption of vegetables, green salad and fruit as well as poultry, rice and pasta. For example, those with high adherence to DP2 consumed on average more than one fruit (180 g) per day, and their consumption of fruits and vegetables was three times higher, compared with those with low adherence. DP3 (6·7 % of total variance) was mainly characterised by high consumption of bread, cheese and marmalade, jam and sugar. The intake of these foods among participants with high adherence to DP3 was twice as high compared with those with low adherence. Positive loadings for potato, meat and egg and negative loading for fermented milk mainly characterised DP4 (6·1 % of total variance). Participants with high adherence to DP4 reported a mean intake of 763 g meat per week, 300 g more than those with low adherence (455 g). On average, there were only small differences in consumption of fruit and vegetables regardless of adherence to DP4.

The mean intake of selected nutrients is displayed in Supplementary Table 6, based on adherence to the respective DP.

### Dietary patterns and sarcopenia

The associations between the DP and sarcopenia (EWGSOP2) are displayed in Table 3. When the DP were used as continuous variables (estimates expressed per 1 sd increment), DP1, DP3 and DP4 had an adjusted OR (model 3) above 1·0 (1·04, 1·19 and 1·08, respectively), with wide CIs. The adjusted OR (model 3) for DP2 and sarcopenia was 0·70 (95 % CI: 0·48, 1·03).

**Table 3.** Logistic regression analysis between adherence to each dietary pattern at baseline (mean age 71) and prevalence of sarcopenia defined according to EWGSOP2 at follow-up (mean age 87) (Odd ratio and 95 % confidence intervals).

	Adherence to dietary pattern						
	Low	Medium		High		Continuous (per 1 sd) increment)	
	OR	OR	95 % CI	OR	95 % CI	OR	95 % CI
DP 1							
Participants, *n*	98	68		91		257	
Sarcopenia							
*n*	17	13		20		50	
%	17	19		22		19	
Model 1*	1·00 (ref)	1·13	0·51, 2·50	1·34	0·65, 2·76	1·11	0·85, 1·46
Model 2†	1·00 (ref)	1·12	0·48, 2·60	1·34	0·59, 3·02	1·13	0·82, 1·56
Model 3‡	1·00 (ref)	1·22	0·49, 3·02	1·13	0·48, 2·70	1·04	0·74, 1·46
DP 2							
Participants, *n*	74	91		92		257	
Sarcopenia							
*n*	20	15		15		50	
%	27	16		16		19	
Model 1*	1·00 (ref)	0·53	0·25, 1·13	0·53	0·25, 1·12	0·79	0·57, 1·11
Model 2†	1·00 (ref)	0·49	0·23, 1·08	0·48	0·22, 1·07	0·76	0·53, 1·07
Model 3‡	1·00 (ref)	0·41	0·17, 0·98	0·40	0·17, 0·94	0·70	0·48, 1·03
DP 3							
Participants, *n*	67	81		109		257	
Sarcopenia							
*n*	17	12		21		50	
%	25	15		19		19	
Model 1*	1·00 (ref)	0·51	0·22, 1·17	0·70	0·34, 1·45	1·06	0·79, 1·42
Model 2†	1·00 (ref)	0·47	0·18, 1·24	0·70	0·21, 1·81	1·41	0·85, 2·32
Model 3‡	1·00 (ref)	0·50	0·18, 1·40	0·53	0·17, 1·70	1·19	0·71, 2·00
DP 4							
Participants, *n*	100	83		74		257	
Sarcopenia							
*n*	16	18		16		50	
%	16	22		22		19	
Model 1*	1·00 (ref)	1·45	0·69, 3·07	1·45	0·67, 3·13	1·04	0·76, 1·41
Model 2†	1·00 (ref)	1·53	0·71, 3·31	1·47	0·66, 3·30	1·05	0·76, 1·47
Model 3‡	1·00 (ref)	1·57	0·69, 3·57	1·61	0·67, 3·87	1·08	0·77, 1·53

DP, dietary pattern.

Participants were categorised as low, medium or high adherent to each DP based on factor scores and according to tertiles limits determined based on adherence to dietary patterns at baseline in the total population of 1133 men.

* Model 1: unadjusted for potential confounders.

† Model 2: adjusted for age at baseline (continuous), follow-up period (continuous), reported energy intake at baseline (continuous), education (categorical), physical activity at baseline (categorical), smoking (categorical) and morbidity at baseline (categorical).

‡ Model 3: further adjusted for BMI at baseline (continuous).

In analyses with the DP as categorical variables medium and high adherence *v*. low adherence; Table 3), the p-values from the Wald tests were 0·91, 0·06, 0·40 and 0·47 for DP1, DP2, DP3 and DP4, respectively, indicating that the association between DP2 and sarcopenia may be nonlinear. Compared with low adherence to DP2, both medium and high adherence was associated with lower OR of sarcopenia, adjusted OR (model 3): 0·41 (95 % CI: 0·17, 0·98) and 0·40 (95 % CI: 0·17, 0·94), respectively. No clear patterns were observed for DP1, DP3 or DP4 in relation to sarcopenia and confidence intervals were wide (Table 3, online Supplementary Fig. 3). Discrepancies between linear and categorical OR for DP3, where the linear OR was 1·19/sd (95 % CI: 0·71, 2·00) and medium and high adherence (*v*. low adherence) had adjusted OR of 0·50 (95 % CI: 0·18, 1·40) and 0·53 (95 % CI: 0·17, 1·70) can be explained by the cut-off limits indicated in Supplementary Fig. 3. A higher adherence to DP4 was associated with higher odds ratio of sarcopenia although CI were wide (OR: 1·61, 95 % CI: 0·67, 3·87, model 3).

No clear associations were observed between DP and sarcopenia, defined according to EWGSOP1 (online Supplementary Table 7).

### Dietary patterns and the variables used in the definition of sarcopenia

**Fig. 1.**
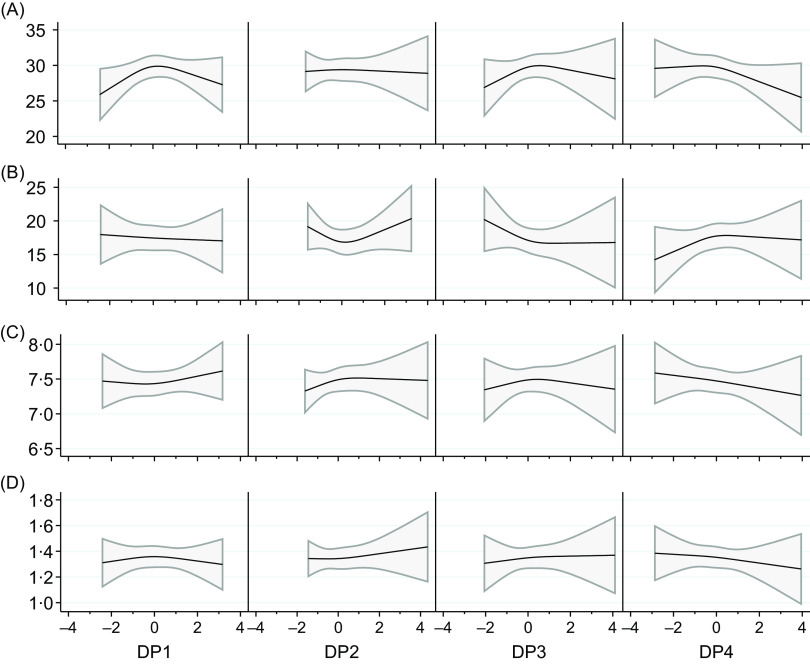
Associations between dietary pattern (DP) 1–4 with the variables used in the definition of sarcopenia: A. hand grip strength (kg), *n* 301, B. chair stand test (seconds), *n* 241, C. appendicular lean mass index (kg/m^2^), *n* 257 and D. gait speed (m/s), *n* 257. Beta estimates (on each Y axis) were modelled using restricted cubic splines (three knots placed at the 10th, 50th and 90th percentiles) and were adjusted for age at baseline (continuous), follow-up period (continuous), reported energy intake at baseline (continuous), education (categorical), physical activity at baseline (categorical), smoking (categorical), morbidity at baseline (categorical) and BMI at baseline (continuous), i.e. Model 3. The solid line represents the beta coefficient and the shaded area represents its 95 % CI.

The analyses of DP in relation to the individual variables used in the definition of sarcopenia displayed no clear associations (Fig. 1 and online Supplementary Table 8). However, higher adherence to DP3 was associated with higher handgrip strength, which was statistically significant if unadjusted, but not if adjusted according to model 2 or 3. Medium adherence to DP4 was associated with lower handgrip strength and slower gait speed, which was statistically significant both unadjusted and adjusted. Medium adherence to DP4 was also associated with longer time to perform chair stand test, which was statistically significant if unadjusted, but not if adjusted according to model 2 or 3.

### Sensitivity analyses

In a subgroup analysis (*n* 247), we excluded participants (*n* 10) with self-reported body weight loss in the past year before the baseline measurement to limit potential influences of recent change of dietary intake due to, for example, undiagnosed disease before baseline. This exclusion did not affect the overall associations between the DP and sarcopenia (EWGSOP2) (online Supplementary Table 9).

In another subgroup analysis (*n* 237), we excluded participants with BMI below 22 at baseline (*n* 20) to limit the potential risk of participants having low muscle mass at baseline. This exclusion did not significantly affect the association between DP and sarcopenia (EWGSOP2), with the exception of wider CI (online Supplementary Table 10).

In addition, we derived DP, using the same method and thresholds as in the main study group (*n* 1133), in a subgroup of adequate energy reporters (*n* 855), according to the Goldberg cut-offs^(52)^, based on individual specific energy requirements. The derived DP and the associations between the DP and sarcopenia (EWGSOP2) were largely similar to our main analysis, however, with larger CI (online Supplementary Material 2).

## Discussion

In this cohort of 71-year-old Swedish men, adherence to a dietary pattern mainly characterised by a high consumption of fruit, vegetables, poultry, rice and pasta (DP2) was associated with lower prevalence of sarcopenia defined according to EWGSOP2, 16 years later. In contrast, a higher adherence to a dietary pattern mainly characterised by a high consumption of potato, meat and egg and low consumption of fermented milk (DP4) was associated with higher prevalence of sarcopenia, although with low precision. These results add potentially important knowledge to the limited literature using dietary patterns rather than nutrients or single foods, to investigate the association between dietary intake and sarcopenia.

Methods used when defining DP are often subdivided into *a priori* and *a posteriori*. Several *a priori* DP are linked to dietary guidelines and thus based on the current state of knowledge, with the limitation that they do not necessarily capture the complexity of the total diet. *A posteriori* defined DP are, in contrast, statistically derived patterns from the dietary data at hand without prior hypotheses about the association between diet and outcome and are to a larger extent population specific. *A posteriori* approaches can, thus, be useful when knowledge of the association between diet and outcome is limited, as with diet and sarcopenia. Factor analysis, including PCA used in the present study, calculates a score for each individual’s adherence to each DP, reflecting that most participants eat all types of foods, albeit in different amounts and frequencies. In cluster analysis, each participant contributes to one single derived cluster. Given that, from a statistical point of view, our study population is small, factor analysis may be preferable.

In PCA-derived DP, all included food groups contribute to each DP and explain the total diet; low or no intake of foods may be as important as foods consumed in larger amounts. We, therefore, label the DP in our study as DP1, DP2, DP3 and DP4, rather than label them as ‘Western’ or ‘Healthy’. As none of the previously published studies includes DP similar to DP1 or DP3, and we do not display any clear associations, these are not discussed further. The DP explained 28 % of the total variance, leaving 72 % of the variance unexplained. This is similar to what has been reported from other studies and may be considered as fairly high explanation rate due to the diversity of dietary habits within a population.

Previous longitudinal studies are few and reported no associations between a posteriori DP and incident sarcopenia in community-dwelling men and women in Hong Kong^(24)^ (age ≥ 65 years, 4-year follow-up) and UK^(53)^ (age ≥ 85 years; 3-year follow-up). However, cross-sectional associations were observed in these, and other, studies. A DP characterised by a high consumption of butter, red meat, gravy, vegetables, sweets/dessert and potatoes was associated with higher prevalence of sarcopenia^(53)^. DP characterised by a high intake of poultry^(54)^; vegetables, fruit, fish, nuts and vegetable oil^(25,26)^; vegetables and fruits^(24)^; snacks, coffee, milk and fast food^(24)^; vegetables, fruit, fish and tofu^(55)^ were all associated with a lower prevalence of sarcopenia. Even though most of previous studies report that high adherence to DP characterised by high intake of vegetables and fruit is associated with lower prevalence of sarcopenia, the total diet seems important, and a high intake of vegetables alone may not be enough as dissonant results have been reported^(23,24,53)^. Dietary patterns add context to the single food items. Dissimilar findings may be explained by regional or cultural differences in how foods are used in cooking or combined in meals, how foods are combined into food groups by the researchers, the definition of sarcopenia (e.g. EWGSOP1 *vs*. EWGSOP2), and follow-up period.

Based on the same cohort as in this study, we displayed that a Mediterranean-like diet was associated with lower prevalence of sarcopenia (EWGSOP1)^(23)^. Despite the similarities of the Mediterranean diet with DP2 in the present study, we did not see an association of DP2 with sarcopenia according to EWGSOP1 in the present study. Although several aspects are similar between DP2 in the current study and the Mediterranean-like diet used in our previous study^(23)^, the factor loading of alcohol in DP2 is low but is included as one item in the Mediterranean-like diet. However, the significance of this is difficult to estimate. The sarcopenia definitions partly capture different individuals and differ mainly in the decisive variables used; the chair stand test in EWGSOP2 (main outcome in the present study) and handgrip strength in EWGSOP1. In the present study, DP2 was associated with sarcopenia defined as the combination of muscle function and muscle mass, but not with the components of sarcopenia. This could be an indication that the combination of an impaired physical function, low muscle mass and poor strength may be more detrimental than each component on its own, similar to the complexity of dietary intake where DP represent a more complete picture than single foods or nutrients. However, a high adherence to DP4 seemed to have an inverse association with muscle function and muscle mass. A similar DP characterised by, among other food groups, high intake of red meat and potatoes was inversely associated with muscle strength and physical performance^(53)^.

In the present study, DP were calculated based on the actual intake of each food group in grams^(21)^ and may represent different energy densities. To ensure that any associations between the DP and the outcome are not due to differences in energy intake, we include energy intake as a covariate in the multivariable analyses and excluded extreme outliers of energy intake. This allows for ranking of individuals’ dietary intakes, which is essential when studying associations^(28)^. Additional exclusion of inadequate energy reporters^(52)^ in a sensitivity analysis is described in Supplementary Material 2. To limit misreporting of dietary intake, we used a valid 7-d menu book (online Supplementary Material 1) that was filled in prospectively and allowed for adequate calculation of total energy intake, however, with potential selection of what is eaten during this specific period. An FFQ would cover a larger period and might provide a broader picture but is on the other hand recorded retrospectively.

Several studies, examining single food groups, have reported a high intake of fruit and vegetables being associated with lower prevalence of sarcopenia^(56)^, in line with our and previous studies on dietary patterns. Mechanisms may involve anti-oxidative^(57)^ and anti-inflammatory^(58)^ effects on skeletal muscle and ageing, potentially involving the phytochemical content of fruits and vegetables^(56)^ or the acid–base balance of the diet, as net acid load has been reported to be associated with loss of muscle mass^(59)^. A high intake of protein has been associated with muscle mass^(60)^, and a higher intake of protein per kg/body weight has been recommended^(61)^. In this context, it is interesting to note that a higher adherence to our DP2 was associated with a higher absolute protein intake (online Supplementary Table 6). However, Granic et al. have reported that a DP (high in butter, red meat, gravy and potato) was associated with an increased risk of sarcopenia, even when the intake of protein was high^(53)^.

Strengths of the present study include the longitudinal approach with a long follow-up period, a well-characterised population, and that we adjust for a large number of potential confounders including BMI and physical activity that are associated with both energy requirement and lifestyle. The relatively small study population was partly compensated for by an age-standardised set-up and a follow-up period well adapted to the period when sarcopenia begins to become more common^(62–65)^. As the age-related decline of muscle mass and function is slow, and the sarcopenia definition is based on thresholds, a long follow-up period is crucial. We created the DP among the full population at baseline, and not only among those attending the follow-up, to better reflect the distribution of the DP at that time. However, it should be borne in mind that the diet among 71-year-old men in the 1990s probably does not correspond to a typical diet in that age group in the 2020s. Dietary patterns are influenced by cultural eating habits and may therefore not be easily transferable to other populations^(21)^. This is somewhat counteracted by the fact that we are ranking individuals according to DP adherence and do not focus on specific amounts of foods or nutrients.

Using a single measure of dietary intake as a reflection of habitual dietary habits may be seen as a limitation as the dietary intake may change over time although adherence to dietary patterns has been reported to be reasonably stable during 10 years in Swedish and other populations^(66–68)^. However, if dietary habits changed during the follow-up period, it would probably affect our estimates towards the null.

The main limitation of the present study is that we were not able to define sarcopenia at baseline. The prevalence of sarcopenia at an approximate age of 70 in this population is usually considered as low^(64)^. We did not see a major impact on our estimates by exclusion of those with a low BMI at baseline, being at higher risk of having sarcopenia. Nonetheless, we cannot preclude the possibility of reverse causation. There may be an influence of residual or unmeasured confounding, and we cannot exclude that any statistically significant associations may have occurred by chance. Because sarcopenia was defined on average 16 years after baseline at a follow-up examination, participants included in our study had a higher physical activity level and fewer comorbidities at baseline, compared to excluded participants, a possible result of a healthy survivor effect or natural selection by increasing age, introducing potential selection bias. However, since many confounders in our adjusted model are linked with reasons for nonparticipation, the influence of such selection bias is limited^(69)^. The participants in our study probably had a normal nutritional status at baseline, even though we lack specific information on nutritional status. Our results may not be generalisable to a more sedentary population, to a population with more comorbidities or with great cultural differences in eating habits, to other age groups, or to women^(17,63-65)^.

### Conclusions

In summary, using an *a posteriori* approach and reported dietary intake in a cohort of community-dwelling 71-year-old Swedish men, we defined four DP. A higher adherence to DP2, mainly characterised by high consumption of vegetables, green salad and fruit as well as poultry, rice and pasta, was associated with a lower prevalence of sarcopenia defined according to EWGSOP2 16 years later. Contradicting results when comparing studies, using different methods, stress the need to use equivalent definitions and methodology when defining sarcopenia. Thus, it could be of value to consider reanalysis of some previous studies, using EWGSOP1 and EWGSOP2 for the diagnosis of sarcopenia.

## Supplementary material

For supplementary material accompanying this paper visit https://doi.org/10.1017/S0007114521003901. click here to view supplementary material
