# Supplementary material for: A posteriori dietary patterns in 71-year-old Swedish men and the prevalence of sarcopenia 16 years later
Source: Br J Nutr. 2021 Sep 29;128(5):909–20. doi: 10.1017/S0007114521003901 (PMC9361200; doi:10.1017/S0007114521003901)
Supplement: Supplementary file 1 [file S0007114521003901sup001.pdf]

Supplementary Table 1
Included food items in each of the food groups

**Food groups**	**Included food items**
Fermented milk	Sour milk and yoghurt. Flavored and unflavored. Sweetened and unsweetened
Milk	Milk including sweet milk drinks
Cereals	Breakfast cereals, oatmeal and porridge
Bread	All kinds of bread including Swedish crispbread
Cheese	Cheese, cream cheese, cottage cheese and cheese-spread
Cold meats	Ham, salami, charcuterie and liver pate
Soup	Soup with meat, fish or vegetables. Excluding traditional pea soup
Pancake	Pancake, pancake with diced pork and crepes
Quiche and pie	Quiche, pie, pizza and pasty
Salad with protein	Salad with protein, mainly chicken or Greek salad
Meat	Meat, processed meat, offal, black pudding and related dishes
Poultry	Poultry and related dishes
Seafood	Fish, seafood and related dishes. Excluding Swedish caviar (sweetened and salted cod roe)
Egg	Egg and related dishes
Salad	Lettuce and cucumber
Vegetables	Vegetables, root vegetables and related dishes. Excluding potato
Legumes	Pulses, lentils, beans, peas and related dishes. Including traditional pea soup
Pasta and rice	All kinds of pasta and rice
Potato	Potatoes and related dishes
Juice	Juice (100% fruit) and nectar (25-50% fruit)
Fruit	Fruit and berries. Fresh and dried
Soft drink	Soda, lemonade and other sweetened drinks
Alcohol content <4%	Beer and cider with alcohol content < 4%
Alcohol content >4%	Beer, cider, wine and spirits with alcohol content > 4%
Marmalade, jam and sugar	All kinds of marmalade, jam and sugar, including honey
Dessert and pastry	Sweet pie, sweet soup, ice-cream, bakery, cookies, buns and canned fruit

Supplementary Table 2

Dietary patterns and principal components loadings for each of the food group

Food groups[a]	DP1	DP2	DP3	DP4
Bread	-0.0338	0.0478	**0.5111**	0.1925
Cheese	-0.0726	-0.0210	**0.4922**	0.0298
Fermented milk	-0.1685	0.0098	0.2717	**-0.3351**
Milk	**0.4505**	0.0211	0.0325	0.1741
Soup	0.0215	0.1643	0.0818	-0.1440
Pancake	0.2604	-0.1222	0.1229	-0.1914
Quiche/pie	-0.0079	-0.0813	0.1400	-0.1241
Salad with protein	-0.2497	0.1842	-0.0010	0.0257
Cold meats	-0.0762	0.0855	0.1413	0.1963
Meat	0.0531	-0.0260	0.1428	**0.4806**
Poultry	-0.0869	**0.3334**	-0.1480	0.0830
Egg	-0.0724	0.0084	-0.0068	**0.3298**
Seafood	-0.0398	0.0654	0.0078	-0.0065
Vegetables	0.0783	**0.4927**	0.0183	0.0090
Green salad	-0.1360	**0.3271**	0.0976	0.0538
Legumes	0.1634	-0.0497	0.0351	0.0402
Juice	-0.0534	0.1750	0.0977	-0.2489
Fruit	0.0621	**0.4513**	0.0715	-0.1088
Pasta and rice	0.0478	**0.4164**	-0.0866	0.0371
Potato	0.0991	0.0347	0.1694	**0.4077**
Cereals	**0.4846**	0.0686	-0.0294	0.0743
Desserts and pastry	0.1844	0.0211	0.2851	-0.2661
Marmalade, jam and sugar	0.1338	-0.0480	**0.3806**	-0.1091
Soft drink	0.0998	0.0856	0.0928	-0.0080
Alcohol <4 %	**-0.3631**	-0.1207	0.1364	0.1599
Alcohol >4 %	**-0.3315**	0.0154	0.0401	0.0294
Proportion of variability explained (%)[b]	7.90	6.87	6.68	6.11

[a]Food groups are defined and exemplified in Supplementary Table 1
[b]Total variance explained by these four derived components equals 27.6%.
Principal components loadings >.30 and <-.30 in bold

Supplementary Table 3

Characteristics at baseline (mean age 71), for the 257 men included in the main analysis with sarcopenia as outcome, the total population at baseline, and for the 876 men who were not included in the main analysis. Values at follow-up (mean age 87) are presented for the population with follow-up information.

		Population included in main analysis<br>n=257	Total population at baseline<br>n=1,133	Population not included in main analysis (no follow-up)<br>n=876
Age (years)	baseline	70.9 (0.6)	71.0 (0.6)	71.0 (0.6)
Follow-up period (years)		15.7 (0.7)		
Body weight (kg)	baseline	79.5 (9.9)	80.4 (11.4)	80.6 (11.8)
	follow-up	76.2 (11.5)		
BMI (kg/m^2) baseline	baseline	25.9 (2.9)	26.3 (3.4)	26.4 (3.5)
	follow-up	25.6 (3.5)		
Appendicular LMI (kg/m^2)	follow-up	7.5 (0.8)		
Chair stand test (s)	follow-up	17.9 (7.3)		
Handgrip strength (kg)	follow-up	30.1 (6.2)		
Gait speed (m/s)	follow-up	1.4 (0.3)		
Reported energy intake (kcal/day)	baseline	1853 (441)	1747 (449)	1716 (447)
Reported protein intake (g/day)	baseline	69 (16)	67 (17)	66 (17)
Reported protein intake (g/kg body weight/day)	baseline	0.88 (0.24)	0.85 (0.25)	0.84 (0.25)
Years of education (n)				
6-7 years		122 (47%)	638 (56%)	516 (59%)
8-13 years		81 (32%)	328 (29%)	247 (28%)
> 13 years		54 (21%)	167 (15%)	113 (13%)
Physical activity level (n)	baseline			
Low		81 (32%)	419 (37%)	338 (39%)
Medium		156 (61%)	611 (54%)	455 (52%)
High		20 (8%)	64 (6%)	44 (5%)
Missing			39 (3%)	39 (4%)
Smoking (n)	baseline			
Never		104 (40%)	325 (29%)	221 (25%)
Current		28 (11%)	225 (20%)	197 (22%)
Former		125 (49%)	583 (51%)	458 (52%)
Charlson Comorbidity Index (n)	baseline			
0		202 (79%)	794 (70%)	592 (68%)
≥1		55 (21%)	339 (30%)	284 (32%)

Values are presented as mean (standard deviation) for continuous measures, and number (percentage) for categorical measures

BMI, body mass index; LMI, lean mass index

Supplementary Table 4

Dietary intake among the full baseline population used for calculation of the dietary patterns (n=1133), displayed as food groups at baseline and by adherence to dietary patterns at baseline

	**Total**	**DP1**			**DP2**			**DP3**			**DP4**		
		**Low**	**Medium**	**High**	**Low**	**Medium**	**High**	**Low**	**Medium**	**High**	**Low**	**Medium**	**High**
**Food groups**[a]	n=1,133	n=378	n=378	n=377	n=378	n=378	n=377	n=378	n=378	n=377	n=378	n=378	n=377
Cheese	34 (22)	37 (22)	33 (23)	33 (21)	34 (23)	32 (19)	37 (23)	**21 (14)**	**33 (17)**	**50 (23)**	33 (20)	33 (21)	37 (24)
Bread	103 (39)	103 (37)	106 (39)	100 (40)	96 (36)	102 (36)	110 (43)	**75 (25)**	**101 (29)**	**132 (38)**	92 (34)	98 (36)	118 (41)
Fermented milk	94 (102)	116 (108)	103 (103)	61 (84)	84 (100)	94 (102)	103 (102)	62 (80)	88 (94)	130 (117)	**146 (112)**	**81 (89)**	**54 (79)**
Milk	243 (194)	**113 (119)**	**226 (143)**	**392 (197)**	245 (191)	241 (192)	244 (198)	225 (186)	238 (184)	267 (209)	204 (168)	230 (170)	297 (225)
Soup	23 (38)	24 (38)	24 (37)	23 (41)	15 (27)	22 (34)	33 (49)	17 (32)	24 (42)	29 (40)	31 (46)	21 (34)	18 (32)
Pancake	20 (23)	11 (18)	19 (20)	29 (26)	25 (24)	18 (21)	17 (21)	16 (20)	21 (23)	23 (25)	26 (24)	20 (22)	14 (21)
Quiche and pie	6 (17)	6 (17)	6 (16)	6 (18)	8 (20)	6 (15)	4 (15)	3 (10)	6 (17)	9 (21)	9 (21)	5 (14)	4 (13)
Salad with protein	6 (15)	13 (21)	5 (12)	1 (5)	3 (9)	5 (11)	12 (21)	6 (15)	7 (15)	7 (15)	6 (15)	6 (14)	7 (17)
Cold meats	14 (13)	15 (13)	15 (14)	12 (12)	11 (11)	14 (13)	17 (15)	11 (10)	14 (13)	18 (15)	11 (11)	13 (12)	19 (16)
Meat	66 (28)	63 (25)	65 (28)	69 (30)	66 (26)	65 (25)	67 (32)	61 (25)	65 (26)	72 (30)	**48 (19)**	**63 (21)**	**86 (28)**
Poultry	5 (9)	7 (9)	5 (9)	4 (7)	**2 (4)**	**4 (7)**	**10 (11)**	6 (10)	5 (8)	4 (8)	4 (8)	5 (9)	6 (9)
Egg	14 (17)	16 (18)	14 (15)	13 (17)	14 (18)	14 (15)	14 (17)	14 (18)	14 (17)	14 (17)	8 (10)	13 (15)	22 (21)
Seafood	4 (8)	4 (8)	4 (8)	3 (7)	3 (6)	4 (8)	5 (9)	3 (8)	4 (7)	4 (9)	4 (8)	4 (8)	4 (8)
Vegetables	23 (23)	22 (20)	23 (21)	25 (26)	**11 (10)**	**20 (14)**	**38 (30)**	20 (21)	23 (23)	26 (24)	23 (22)	21 (20)	26 (25)
Green salad	33 (35)	42 (38)	31 (36)	26 (28)	**16 (18)**	**29 (25)**	**53 (45)**	25 (29)	32 (33)	41 (40)	30 (32)	32 (36)	36 (36)
Legumes	16 (24)	10 (19)	16 (24)	23 (27)	20 (26)	15 (23)	15 (24)	15 (24)	16 (23)	19 (26)	15 (23)	15 (22)	19 (27)
Juice	25 (55)	31 (63)	26 (55)	18 (43)	10 (28)	22 (45)	43 (75)	16 (38)	25 (56)	34 (66)	42 (77)	19 (39)	13 (34)
Fruit	118 (102)	113 (98)	120 (101)	122 (106)	**58 (56)**	**114 (76)**	**183 (120)**	100 (101)	117 (98)	138 (103)	135 (100)	110 (107)	109 (97)
Pasta and rice	18 (24)	18 (21)	18 (23)	19 (29)	**8 (10)**	**15 (17)**	**32 (33)**	20 (28)	16 (21)	18 (24)	18 (22)	16 (20)	21 (30)
Potato	141 (61)	131 (56)	143 (61)	150 (63)	137 (49)	139 (58)	148 (72)	123 (50)	143 (56)	159 (69)	**109 (45)**	**139 (46)**	**176 (68)**
Cereals	80 (87)	**23 (33)**	**60 (59)**	**158 (91)**	76 (83)	83 (88)	82 (89)	75 (85)	84 (88)	82 (86)	74 (80)	73 (84)	94 (94)
Dessert and pastry	77 (70)	53 (47)	79 (68)	99 (82)	68 (66)	81 (76)	81 (66)	48 (52)	75 (62)	108 (79)	102 (77)	69 (64)	59 (60)
Marmalade, jam and sugar	21 (18)	17 (16)	20 (16)	27 (20)	22 (17)	21 (19)	21 (18)	**13 (11)**	**19 (13)**	**32 (23)**	24 (19)	20 (17)	20 (18)
Soft drink	39 (91)	24 (73)	44 (99)	49 (99)	30 (71)	41 (83)	46 (114)	24 (59)	41 (100)	53 (105)	40 (93)	36 (88)	41 (93)
Alcohol content <4%	148 (151)	238 (181)	124 (117)	82 (94)	165 (176)	150 (144)	129 (125)	119 (152)	151 (147)	174 (149)	114 (120)	149 (148)	182 (172)
Alcohol content >4%	28 (56)	57 (81)	17 (31)	9 (23)	25 (47)	27 (62)	31 (58)	26 (46)	27 (64)	30 (56)	25 (41)	33 (73)	25 (49)

Values are presented as mean daily intake in gram (standard deviation)

Food groups with principal components loadings >.30 and <-.30 in bold

[a]Food groups are defined and exemplified in Supplementary Table 1

Supplementary Table 5

Characteristics at baseline (mean age 71) and follow-up (mean age 87) of men included in the main analysis and grouped according to adherence to each dietary pattern at baseline.

			DP1			DP2			DP3			DP4		
		**Total**	**Low**	**Medium**	**High**	**Low**	**Medium**	**High**	**Low**	**Medium**	**High**	**Low**	**Medium**	**High**
		n=257	n=98	n=68	n=91	n=74	n=91	n=92	n=67	n=81	n=109	n=100	n=83	n=74
Age (years)	baseline	70.9 (0.6)	71.0 (0.6)	70.8 (0.6)	71.0 (0.6)	71.0 (0.6)	71.0 (0.6)	70.9 (0.6)	70.9 (0.6)	70.9 (0.6)	71.0 (0.6)	70.9 (0.6)	71.0 (0.5)	70.9 (0.7)
Follow-up period (years)		15.7 (0.7)	15.6 (0.7)	15.7 (0.6)	15.6 (0.7)	15.7 (0.7)	15.7 (0.6)	15.6 (0.7)	15.6 (0.6)	15.6 (0.7)	15.7 (0.7)	15.6 (0.7)	15.8 (0.7)	15.6 (0.6)
Body weight (kg)	baseline	79.5 (9.9)	80.6 (9.9)	80.2 (8.3)	77.8 (10.9)	80.0 (9.6)	79.7 (11.3)	78.9 (8.7)	80.6 (12.5)	80.1 (8.8)	78.4 (8.9)	80.0 (10.6)	78.9 (9.7)	79.5 (9.4)
Body weight (kg)	follow-up	76.2 (11.5)	76.5 (11.0)	77.8 (9.9)	74.7 (13.2)	76.0 (13.3)	76.3 (11.5)	76.4 (10.1)	75.7 (13.0)	76.9 (12.2)	76.0 (10.1)	76.9 (12.5)	75.5 (11.3)	76.1 (10.6)
BMI (kg/m^2)	baseline	25.9 (2.9)	26.0 (2.9)	26.4 (2.7)	25.4 (3.0)	26.1 (3.0)	26.0 (3.0)	25.6 (2.6)	26.2 (3.5)	26.3 (2.8)	25.4 (2.3)	25.9 (3.0)	25.8 (2.8)	25.9 (2.8)
BMI (kg/m^2)	follow-up	25.6 (3.5)	25.5 (3.3)	26.3 (3.1)	25.2 (4.0)	25.7 (4.4)	25.6 (3.1)	25.5 (3.1)	25.4 (3.9)	26.1 (4.0)	25.3 (2.9)	25.7 (3.7)	25.5 (3.4)	25.6 (3.4)
Appendicular LMI (kg/m^2)	follow-up	7.5 (0.8)	7.4 (0.8)	7.5 (0.7)	7.5 (0.8)	7.4 (0.9)	7.5 (0.7)	7.5 (0.6)	7.4 (0.8)	7.5 (0.8)	7.5 (0.7)	7.5 (0.8)	7.4 (0.8)	7.4 (0.7)
Reported energy intake (kcal/day)	baseline	1853 (441)	1700 (387)	1853 (414)	2018 (459)	1744 (423)	1822 (415)	1972 (456)	1407 (248)	1820 (302)	2152 (376)	1813 (407)	1807 (442)	1959 (470)
Reported protein intake (g/day)	baseline	69 (16)	63 (13)	70 (15)	74 (17)	64 (14)	67 (15)	74 (18)	55 (10)	67 (11)	79 (15)	65 (14)	67 (15)	76 (18)
Reported protein intake (g/kg body weight/day)	baseline	0.88 (0.24)	0.80 (0.20)	0.88 (0.22)	0.97 (0.27)	0.81 (0.20)	0.87 (0.25)	0.95 (0.25)	0.70 (0.18)	0.84 (0.19)	1.02 (0.23)	0.83 (0.19)	0.87 (0.25)	0.98 (0.27)
Years of education (n)														
6-7 years		122 (47%)	29 (30%)	37 (54%)	56 (62%)	37 (50%)	49 (54%)	36 (39%)	37 (55%)	40 (49%)	45 (41%)	39 (39%)	39 (47%)	44 (59%)
8-13 years		81 (32%)	34 (35%)	21 (31%)	26 (29%)	24 (32%)	27 (30%)	30 (33%)	16 (24%)	25 (31%)	40 (37%)	33 (33%)	27 (33%)	21 (28%)
> 13 years		54 (21%)	35 (36%)	10 (15%)	9 (10%)	13 (18%)	15 (16%)	26 (28%)	14 (21%)	16 (20%)	24 (22%)	28 (28%)	17 (20%)	9 (12%)
Physical activity level (n)	baseline													
Low		81 (32%)	34 (35%)	17 (25%)	30 (33%)	20 (27%)	33 (36%)	28 (30%)	27 (40%)	21 (26%)	33 (30%)	32 (32%)	30 (36%)	19 (26%)
Medium		156 (61%)	54 (55%)	44 (65%)	58 (64%)	51 (69%)	49 (54%)	56 (61%)	37 (55%)	52 (64%)	67 (61%)	57 (57%)	47 (57%)	52 (70%)
High		20 (8%)	10 (10%)	7 (10%)	3 (3%)	3 (4%)	9 (10%)	8 (9%)	3 (4%)	8 (10%)	9 (8%)	11 (11%)	6 (7%)	3 (4%)
Smoking (n)	baseline													
Never		104 (40%)	41 (42%)	25 (37%)	38 (42%)	29 (39%)	37 (41%)	38 (41%)	15 (22%)	40 (49)	49 (54)	43 (43%)	35 (42%)	26 (35%)
Current		28 (11%)	11 (11%)	6 (9%)	11 (12%)	8 (11%)	14 (15%)	6 (7%)	17 (25%)	6 (7%)	5 (5%)	11 (11%)	9 (11%)	8 (11%)
Former		125 (49%)	46 (47%)	37 (54%)	42 (46%)	37 (50%)	40 (44%)	48 (52%)	35 (52%)	35 (43%)	55 (50%)	46 (46%)	39 (47%)	40 (54%)
Charlson Comorbidity Index (n)	baseline													
0		202 (79%)	77 (79%)	52 (76%)	73 (80%)	60 (81%)	70 (77%)	72 (78%)	52 (78%)	64 (79%)	86 (79%)	77 (77%)	61 (73%)	64 (86%)
≥1		55 (21%)	21 (21%)	16 (24%)	18 (20%)	14 (19%)	21 (23%)	20 (22%)	15 (22%)	17 (21%)	23 (21%)	23 (23%)	22 (27%)	10 (14%)

Values are presented as mean (standard deviation) for continuous measures, and number (percentage) for categorical measures.
BMI, body mass index; LMI, lean mass index;

Supplementary Table 6

Intake of nutrients for the 257 men included in the main with sarcopenia (EWGSOP2) as outcome. Displayed according to adherence to respective dietary patterns at baseline.

		**DP1**			**DP2**			**DP3**			**DP4**		
	**Total**	**Low**	**Medium**	**High**	**Low**	**Medium**	**High**	**Low**	**Medium**	**High**	**Low**	**Medium**	**High**
	n=257	n=98	n=68	n=91	n=74	n=91	n=92	n=67	n=81	n=109	n=100	n=83	n=74
Energy intake (kcal/day)	1853 (441)	1700 (387)	1853 (414)	2018 (459)	1744 (423)	1822 (415)	1972 (456)	1407 (248)	1820 (302)	2152 (376)	1813 (407)	1807 (442)	1959 (470)
Carbohydrates (E%)	49 (5)	47 (5)	49 (5)	51 (5)	47 (6)	49 (5)	50 (5)	47 (6)	49 (5)	50 (5)	51 (5)	48 (5)	47 (5)
Fat (E%)	35 (5)	36 (5)	35 (4)	33 (5)	36 (6)	35 (4)	33 (5)	35 (6)	35 (5)	34 (5)	33 (5)	35 (5)	36 (5)
Saturated fat (E%)	15.0 (2.9)	15.3 (3.0)	15.1 (2.5)	14.6 (3.2)	16.1 (3.4)	14.6 (2.4)	14.5 (2.9)	15.0 (2.9)	15.1 (3.4)	15.0 (2.8)	14.6 (2.8)	15.2 (3.1)	15.4 (2.9)
Monounsaturated fat (E%)	12.1 (2.0)	12.5 (2.1)	12.2 (1.8)	11.4 (1.8)	12.6 (2.1)	12.2 (1.8)	11.5 (2.0)	12.1 (2.0)	12.5 (2.3)	12.2 (1.8)	11.4 (1.9)	12.3 (2.0)	12.7 (1.8)
Polyunsaturated fat (E%)	5.2 (1.1)	5.4 (1.2)	5.2 (1.0)	4.9 (1.1)	5.1 (1.1)	5.4 (1.2)	4.9 (1.0)	5.2 (1.1)	5.1 (1.2)	5.2 (1.0)	4.9 (1.0)	5.3 (1.2)	5.3 (1.1)
α-Linolenic acid (g/day)	1 (0)	1 (0)	1 (0)	1 (0)	1 (0)	1 (0)	1 (0)	1 (0)	1 (0)	1 (0)	1 (0)	1 (0)	1 (0)
Protein (E%)	15 (2)	15 (2)	16 (2)	15 (2)	15 (2)	15 (2)	15 (2)	16 (2)	15 (2)	15 (2)	15 (2)	15 (2)	16 (2)
Protein intake (g/day)	69 (16)	63 (13)	70 (15)	74 (17)	64 (14)	67 (15)	74 (18)	55 (10)	67 (11)	79 (15)	65 (14)	67 (15)	76 (18)
Dietary fibre (g/day)	18 (5)	16 (4)	18 (5)	20 (6)	15 (4)	18 (5)	21 (6)	13 (3)	18 (5)	21 (5)	18 (5)	17 (5)	19 (6)
Folic acid (ug/day)	202 (55)	192 (48)	202 (51)	214 (62)	168 (37)	191 (44)	241 (54)	155 (31)	199 (47)	234 (50)	205 (55)	191 (48)	212 (61)

Values are presented as mean daily intake (standard deviation)
E%, percent of energy

Supplementary Table 7

Logistic regression analysis between adherence to each dietary pattern at baseline (mean age 71) and prevalence of sarcopenia defined according to EWGSOP1 at follow-up (mean age 87).

	Adherence to dietary pattern			
	Low	Medium	High	Continuous (per 1 SD increment)
		DP 1		
Participants, n	96	68	91	255
Sarcopenia, n	22	14	18	54
OR (95% CI), model 1[a]	1.00 (ref)	0.87 (0.41 - 1.86)	0.83 (0.41 - 1.67)	0.98 (0.75 - 1.27)
OR (95% CI), model 2[b]	1.00 (ref)	0.88 (0.39 - 1.98)	0.80 (0.36 - 1.75)	0.98 (0.72 - 1.35)
OR (95% CI), model 3[c]	1.00 (ref)	0.86 (0.36 - 2.04)	0.60 (0.26 - 1.40)	0.89 (0.64 - 1.24)
		DP 2		
Participants, n	72	91	92	255
Sarcopenia, n	15	19	20	54
OR (95% CI), model 1[a]	1.00 (ref)	1.00 (0.47 - 2.15)	1.06 (0.50 - 2.24)	0.99 (0.73 - 1.34)
OR (95% CI), model 2[b]	1.00 (ref)	1.00 (0.44 - 2.11)	1.16 (0.52 - 2.57)	0.99 (0.73 - 1.36)
OR (95% CI), model 3[c]	1.00 (ref)	0.93 (0.40 - 2.16)	1.05 (0.45 - 2.43)	0.94 (0.67 - 1.31)
		DP 3		
Participants, n	66	81	108	255
Sarcopenia, n	20	12	20	54
OR (95% CI), model 1[a]	1.00 (ref)	**0.40 (0.18 - 0.90)**	0.59 (0.29 - 1.19)	0.89 (0.66 - 1.20)
OR (95% CI), model 2[b]	1.00 (ref)	**0.38 (0.15 - 0.99)**	0.55 (0.19 - 1.59)	1.08 (0.65 - 1.79)
OR (95% CI), model 3[c]	1.00 (ref)	0.37 (0.13 - 1.01)	0.44 (0.14 - 1.35)	0.88 (0.52 - 1.49)
		DP 4		
Participants, n	99	83	73	255
Sarcopenia, n	18	19	17	54
OR (95% CI), model 1[a]	1.00 (ref)	1.34 (0.69 - 3.07)	1.37 (0.65 - 2.88)	0.99 (0.73 - 1.33)
OR (95% CI), model 2[b]	1.00 (ref)	1.31 (0.71 - 3.31)	1.49 (0.70 - 3.27)	1.01 (0.73 - 1.41)
OR (95% CI), model 3[c]	1.00 (ref)	1.31 (0.59 – 2.91)	1.72 (0.74 - 4.02)	1.07 (0.76 - 1.51)

EWGSOP, the European Working Group on Sarcopenia in Older People; DP, dietary pattern; OR, odds ratio; CI, confidence interval; SD, standard deviation.

Statistical significant results in bold

[a] Model 1: unadjusted for potential confounders

[b] Model 2: adjusted for age at baseline (continuous), follow-up period (continuous), reported energy intake at baseline (continuous), education (categorical), physical activity at baseline (categorical), smoking (categorical) and morbidity at baseline (categorical)

[c] Model 3: further adjusted for BMI at baseline (continuous)

Supplementary Table 8

Association of adherence to dietary patterns with functional measures and appendicular lean mass index.

**DP 1**	**Low adherence** β coefficient (95% CI)	**Medium adherence** β coefficient (95% CI)	**High adherence** β coefficient (95% CI)	**Continuous** (per 1 SD increment) β coefficient (95% CI)
**Hand grip strength** (kg)	n=111	n=87	n=103	n=301
Model 1[a]	0 (ref)	-0.317(-2.144 - 1.510)	0.830 (-0.910 - 2.570)	0.416 (-0.269 - 1.102)
Model 2[b]	0 (ref)	-0.156 (-2.067 - 1.756)	0.717 (-1.195 - 2.629)	0.358 (-0.407 - 1.122)
Model 3[c]	0 (ref)	-0.153 (-2.068 - 1.761)	0.678 (-1.247 - 2.604)	0.342 (-0.427 - 1.111)
**Chair stand test** (s)	n=94	n=61	n=86	n=241
Model 1[a]	0 (ref)	1.012 (-1.316 - 3.340)	0.390 (-1.723 - 2.502)	-0.165 (-0.997 - 0.667)
Model 2[b]	0 (ref)	1.210 (-1.090 - 3.510)	0.234 (-1.937 - 2.405)	-0.267 (-1.147 - 0.612)
Model 3[c]	0 (ref)	1.192 (-1.118 - 3.503)	0.255 (-1.929 - 2.438)	-0.256 (-1.141 - 0.629)
**Appendicular LMI** (kg/m^2)	n=98	n=68	n=91	n=257
Model 1[a]	0 (ref)	0.119 (-0.121 - 0.359)	0.027 (-0.194 - 0.248)	0.018 (-0.067 - 0.103)
Model 2[b]	0 (ref)	0.077 (-0.176 - 0.328)	-0.052 (-0.298 - 0.195)	-0.011 (-0.107 - 0.086)
Model 3[c]	0 (ref)	0.033 (-0.181 - 0.246)	0.038 (-0.172 - 0.248)	0.029 (-0.053 - 0.111)
**Gait speed** (m/s)	n=97	n=68	n=92	n=257
Model 1[a]	0 (ref)	0.004 (-0.095 - 0.104)	0.006 (-0.086 - 0.097)	-0.001 (-0.036 - 0.034)
Model 2[b]	0 (ref)	0.017 (-0.086 - 0.120)	0.032 (-0.068 - 0.132)	0.008 (-0.031 - 0.047)
Model 3[c]	0 (ref)	0.015 (-0.086 - 0.117)	0.015 (-0.085 - 0.115)	0.003 (-0.036 - 0.041)

**DP 2**	**Low adherence** β coefficient (95% CI)	**Medium adherence** β coefficient (95% CI)	**High adherence** β coefficient (95% CI)	**Continuous** (per 1 SD increment) β coefficient (95% CI)
**Hand grip strength** (kg)	n=85	n=106	n=110	n=301
Model 1[a]	0 (ref)	0.915 (-0.939 - 2.768)	0.813 (-1.029 - 2.655)	0.076 (-0.677 - 0.830)
Model 2[b]	0 (ref)	0.951 (-0.898 - 2.799)	0.140 (-1.741 - 2.021)	0.050 (-0.731 - 0.831)
Model 3[c]	0 (ref)	0.945 (-0.906 - 2.800)	0.128 (-1.756 - 2.012)	0.038 (-0.745 - 0.821)
**Chair stand test** (s)	n=64	n=92	n=85	n=241
Model 1[a]	0 (ref)	-2.177 (-4.468 - 0.114)	-1.108 (-3.438 - 1.221)	-0.067 (-1.056 - 0.922)
Model 2[b]	0 (ref)	-1.867 (-4.046 – 0.311)	-0.391 (-2.637 - 1.855)	0.282 (-0.677 - 1.242)
Model 3[c]	0 (ref)	-1.859 (-4.043 - 0.325)	-0.382 (-2.633 - 1.869)	0.283 (-0.679 - 1.245)
**Appendicular LMI** (kg/m^2)	n74	n91	n92	n=257
Model 1[a]	0 (ref)	0.175 (-0.062 - 0.412)	0.087 (-0.150 - 0.323)	0.008 (-0.086 - 0.103)
Model 2[b]	0 (ref)	0.147 (-0.095 - 0.389)	0.063 (-0.182 - 0.308)	0.011 (-0.088 - 0.109)
Model 3[c]	0 (ref)	0.162 (-0.043 - 0.367)	0.106 (-0.101 - 0.314)	0.032 (-0.052 - 0.116)
**Gait speed** (m/s)	n=72	n=92	n=93	n=257
Model 1[a]	0 (ref)	0.048 (-0.051 - 0.147)	0.031 (-0.068 - 0.129)	0.023 (-0.017 - 0.063)
Model 2[b]	0 (ref)	0.044 (-0.056 - 0.143)	0.012 (-0.088 - 0.113)	0.016 (-0.025 - 0.057)
Model 3[c]	0 (ref)	0.040 (-0.058 - 0.138)	0.009 (-0.091 - 0.109)	0.013 (-0.028 - 0.053)

DP, dietary pattern; CI, confidence interval; SD, standard deviation; LMI, lean mass index
Statistical significant results in bold
[a] Model 1: unadjusted for potential confounders
[b] Model 2: adjusted for age at baseline (continuous), follow-up period (continuous), reported energy intake at baseline (continuous), education (categorical), physical activity at baseline (categorical), smoking (categorical) and morbidity at baseline (categorical)
[c] Model 3: further adjusted for BMI at baseline (continuous)

Supplementary Table 8 Continued

**DP 3**	**Low adherence** β coefficient (95% CI)	**Medium adherence** β coefficient (95% CI)	**High adherence** β coefficient (95% CI)	**Continuous** (per 1 SD increment) β coefficient (95% CI)
**Hand grip strength** (kg)	n=83	n=96	n=122	n=301
Model 1[a]	0 (ref)	**2.037 (0.144 - 3.931)**	**2.428 (0.630 - 4.227)**	**0.953 (0.244 - 1.662)**
Model 2[b]	0 (ref)	1.666 (-0.520 - 3.852)	1.552 (-1.039 - 4.143)	0.266 (-0.926 - 1.457)
Model 3[c]	0 (ref)	1.659 (-0.530 - 3.848)	1.513 (-1.087 - 4.113)	0.234 (-0.968 - 1.435)
**Chair stand test** (s)	n=64	n=81	n=96	n=241
Model 1[a]	0 (ref)	-0.501 (-2.869 - 1.866)	0.474 (-1.811 - 2.759)	-0.249 (-1.151 - 0.652)
Model 2[b]	0 (ref)	-0.238 (-2.853 - 2.377)	0.760 (-2.306 - 3.827)	-0.449 (-1.829 - 0.931)
Model 3[c]	0 (ref)	-0.257 (-2.879 - 2.364)	0.766 (-2.306 - 3.839)	-0.431 (-1.820 - 0.959)
**Appendicular LMI** (kg/m^2)	n=67	n=81	n=109	n=257
Model 1[a]	0 (ref)	0.150 (-0.101 - 0.400)	0.136 (-0.099 - 0.372)	0.030 (-0.062 - 0.122)
Model 2[b]	0 (ref)	0.125 (-0.165 - 0.415)	0.037 (-0.302 - 0.375)	-0.094 (-0.248 - 0.060)
Model 3[c]	0 (ref)	0.060 (-0.186 - 0.306)	0.104 (-0.183 - 0.392)	0.002 (-0.130 - 0.134)
**Gait speed** (m/s)	n=67	n=81	n=109	n=257
Model 1[a]	0 (ref)	0.033 (-0.071 - 0.137)	0.017 (-0.081 - 0.114)	0.012 (-0.027 - 0.050)
Model 2[b]	0 (ref)	0.043 (-0.075 - 0.162)	0.040 (-0.099 - 0.178)	0.020 (-0.043 - 0.082)
Model 3[c]	0 (ref)	0.044 (-0.073 - 0.161)	0.026 (-0.111 - 0.162)	0.007 (-0.055 - 0.070)

**DP 4**	**Low adherence** β coefficient (95% CI)	**Medium adherence** β coefficient (95% CI)	**High adherence** β coefficient (95% CI)	**Continuous** (per 1 SD increment**)** β coefficient (95% CI)
**Hand grip strength** (kg)	n=111	n=99	n=91	n301
Model 1[a]	0 (ref)	**-2.099 (-3.851 - -0.348)**	-1.109 (-2.895 - 0.678)	-0.452 (-1.187 - 0.284)
Model 2[b]	0 (ref)	**-1.899 (-3.622 - -0.135)**	-1.345 (-3.190 - 0.500)	-0.563 (-1.313 - 0.186)
Model 3[c]	0 (ref)	**-1.892 (-3.658 - -0.125)**	-1.335 (-3.183 - 0.513)	-0.553 (-1.305 - 0.199)
**Chair stand test** (s)	n=95	n=79	n=67	n=241
Model 1[a]	0 (ref)	**2.850 (0.721 - 4.978)**	1.402 (-0.827 - 3.632)	0.528 (-0.387 - 1.444)
Model 2[b]	0 (ref)	1.794 (-0.261 - 3.848)	1.449 (-0.738 - 3.636)	0.399 (-0.500 - 1.299)
Model 3[c]	0 (ref)	1.825 (-0.237 - 3.888)	1.461 (-0.731 - 3.652)	0.398 (-0.503 - 1.299)
**Appendicular LMI** (kg/m^2)	n=100	n=83	n=74	n=257
Model 1[a]	0 (ref)	-0.105 (-0.330 - 0.121)	-0.085 (-0.318 - 0.148)	-0.009 (-0.104 - 0.086)
Model 2[b]	0 (ref)	-0.143 (-0.372 - 0.085)	-0.133 (-0.374 - 0.108)	-0.036 (-0.135 - 0.063)
Model 3[c]	0 (ref)	-0.102 (-0.295 - 0.092)	-0.125 (-0.329 - 0.079)	-0.050 (-0.133 - 0.034)
**Gait speed** (m/s)	n=98	n=84	n=75	n=257
Model 1[a]	0 (ref)	**-0.126 (-0.219 - -0.034)**	-0.081 (-0.177 - 0.014)	-0.027 (-0.066 - 0.012)
Model 2[b]	0 (ref)	**-0.098 (-0.191 - -0.005)**	-0.067 (-0.164 - 0.031)	-0.020 (-0.061 - 0.020)
Model 3[c]	0 (ref)	**-0.098 (-0.190 - -0.007)**	-0.065 (-0.161 - 0.032)	-0.018 (-0.058 - 0.022)

DP, dietary pattern; CI, confidence interval; SD, standard deviation; LMI, lean mass index
Statistical significant results in bold
[a] Model 1: unadjusted for potential confounders
[b] Model 2: adjusted for age at baseline (continuous), follow-up period (continuous), reported energy intake at baseline (continuous), education (categorical), physical activity at baseline (categorical), smoking (categorical) and morbidity at baseline (categorical)
[c] Model 3: further adjusted for BMI at baseline (continuous)

Supplementary Table 9

Logistic regression analysis between adherence to each dietary pattern at baseline (mean age 71) and prevalence of sarcopenia defined according to EWGSOP2 at follow-up (mean age 87). Participants with BMI below 22 at baseline excluded (n= 20).

	Adherence to dietary pattern			
	Low	Med	High	Continuous (per 1 SD increment)
DP 1				
Participants	n90	n66	n81	n237
OR (95% CI), model 3	1.00 (ref)	1.41 (0.54 - 3.67)	1.09 (0.42 - 2.86)	1.02 (0.70 - 1.48)
DP 2				
Participants	n69	n83	n85	n237
OR (95% CI), model 3	1.00 (ref)	0.42 (0.16 - 1.07)	0.52 (0.21 - 1.29)	0.73 (0.50 - 1.07)
DP 3				
Participants	n61	n75	n101	n237
OR (95% CI), model 3	1.00 (ref)	0.88 (0.28 - 2.78)	0.78 (0.22 - 2.81)	1.21 (0.67 - 2.18)
DP 4				
Participants	n92	n76	n69	n237
OR (95% CI), model 3	1.00 (ref)	1.77 (0.70 - 4.50)	1.94 (0.75 – 5.00)	1.20 (0.81 - 1.76)

EWGSOP, the European Working Group on Sarcopenia in Older People; DP, dietary pattern; OR, odds ratio; CI, confidence interval; SD, standard deviation.
Model 3: adjusted for age at baseline (continuous), follow-up period (continuous), reported energy intake at baseline (continuous), education (categorical), physical activity at baseline (categorical), smoking (categorical),morbidity at baseline (categorical) and BMI at baseline (continuous).

Supplementary Table 10

Logistic regression analysis between adherence to each dietary pattern at baseline (mean age 71) and prevalence of sarcopenia defined according to EWGSOP2 at follow-up (mean age 87). Participants who self-reported a reduction in body weight in the last year before baseline measurement excluded (n=10).

	Adherence to dietary pattern			Continuous (per 1 SD increment)
	Low	Med	High	
DP 1				
Participants	n94	n66	n87	n247
OR (95% CI), model 3	1.00 (ref)	1.27 (0.51 - 3.16)	1.15 (0.47 - 2.77)	1.04 (0.73 - 1.47)
DP 2				
Participants	n71	n88	n88	n247
OR (95% CI), model 3	1.00 (ref)	0.43 (0.18 - 1.02)	**0.40 (0.17 - 0.96)**	**0.64 (0.43 – 0.96)**
DP 3				
Participants	n61	n77	n109	n247
OR (95% CI), model 3	1.00 (ref)	0.48 (0.16 - 1.44)	0.53 (0.16 - 1.75)	1.16 (0.68 – 1.98)
DP 4				
Participants	n96	n80	n71	n247
OR (95% CI), model 3	1.00 (ref)	1.86 (0.80 - 4.32)	1.71 (0.69 - 4.21)	1.10 (0.78 - 1.57)

EWGSOP, the European Working Group on Sarcopenia in Older People; DP, dietary pattern; OR, odds ratio; CI, confidence interval; SD, standard deviation.

Statistical significant results in bold

Model 3: adjusted for age at baseline (continuous), follow-up period (continuous), reported energy intake at baseline (continuous), education (categorical), physical activity at baseline (categorical), smoking (categorical), morbidity at baseline (categorical) and BMI at baseline (continuous).

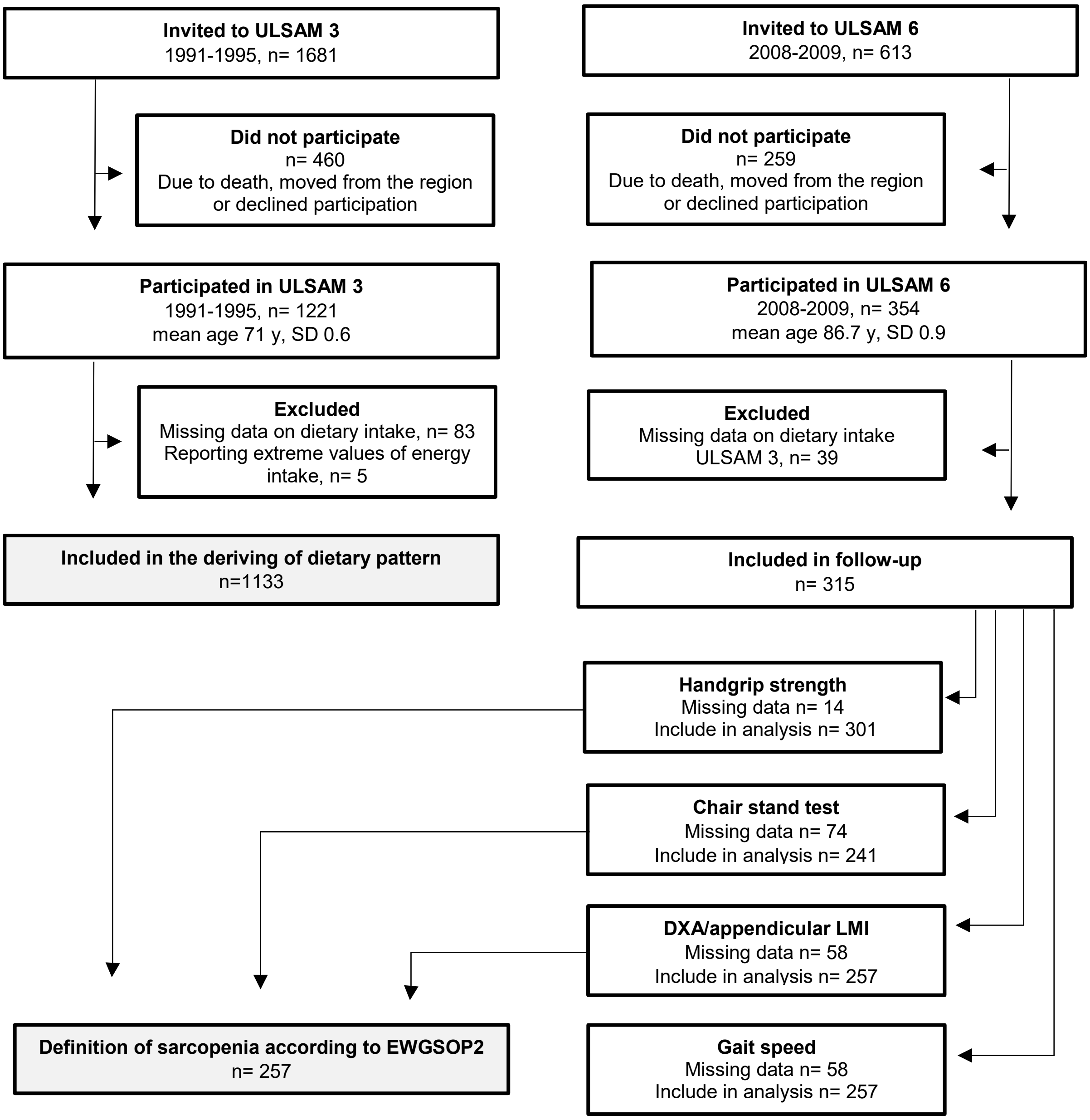

Supplementary Figure 1

Flow chart of invited, excluded and included participants in the study population.
ULSAM, Uppsala Longitudinal Study of Adult Men; SD, standard deviation; DXA, Dual-energy X-ray Absorptiometry; LMI, lean mass index, EWGSOP, the European Working Group on Sarcopenia in Older People

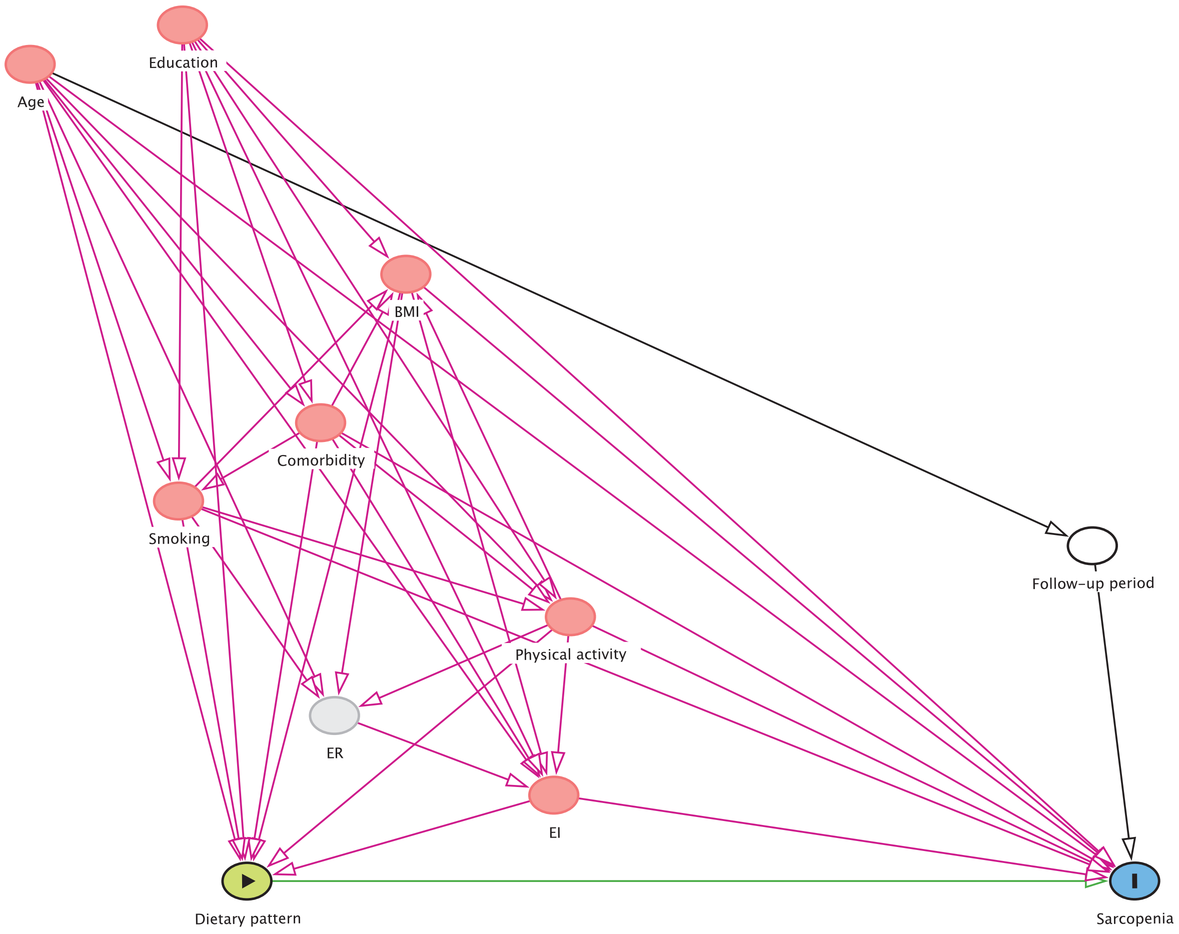

Supplementary Figure 2

A directed acyclic graph approach was applied using DAGitty (dagitty.net)[a] to identify potential confounders to be included in the multivariable models. In this graph, we indicate that *Energy requirements (ER)* is an unobserved variable. We have also added that we want to adjust for *Follow-up period*. Based on this graph, *Age, Energy intake (EI), Education, Physical activity, Smoking, Comorbidity, BMI*, together with the pre-specified variable *Follow-up period*, were suggested as a minimal sufficient adjustment set for estimating the total effect of Dietary pattern on Sarcopenia. This minimal sufficient adjustment set is identical to our model 3.

In a DAG, assumed causal effects are symbolized by directed arrows between the variables, pointing from cause to result. The choice of variables and the direction of the arrows are based on the biological background and the understanding of the potential effects. We assume that *BMI* measured at baseline, at the same time as the dietary assessment, will influence the individual's dietary intake (indicated by the arrow from *BMI* to *Dietary pattern*). It can, however, be assumed that if dietary consumption is maintained over a longer period before and up until our dietary assessment, it may influence *BMI*. This is why we present two adjusted models, one where BMI is not included (model 2) and one where BMI is included (model 3). As to the direction of arrow between *Smoking* and *Comorbidity*, this is also affected by the time aspect where we assume that a previous disease affects current smoking habits. However, changing the direction of this arrow does not alter the minimal sufficient adjustment set indicating what variables to include as confounders.

All variables, except *Follow-up period* and *Sarcopenia*, are based on data at baseline.

ER, Energy requirements; EI, Energy intake; BMI, Body mass index

1. Textor, J., J. Hardt, and S. Knüppel, DAGitty: a graphical tool for analyzing causal diagrams. Epidemiology, 2011. 22(5): p. 745.

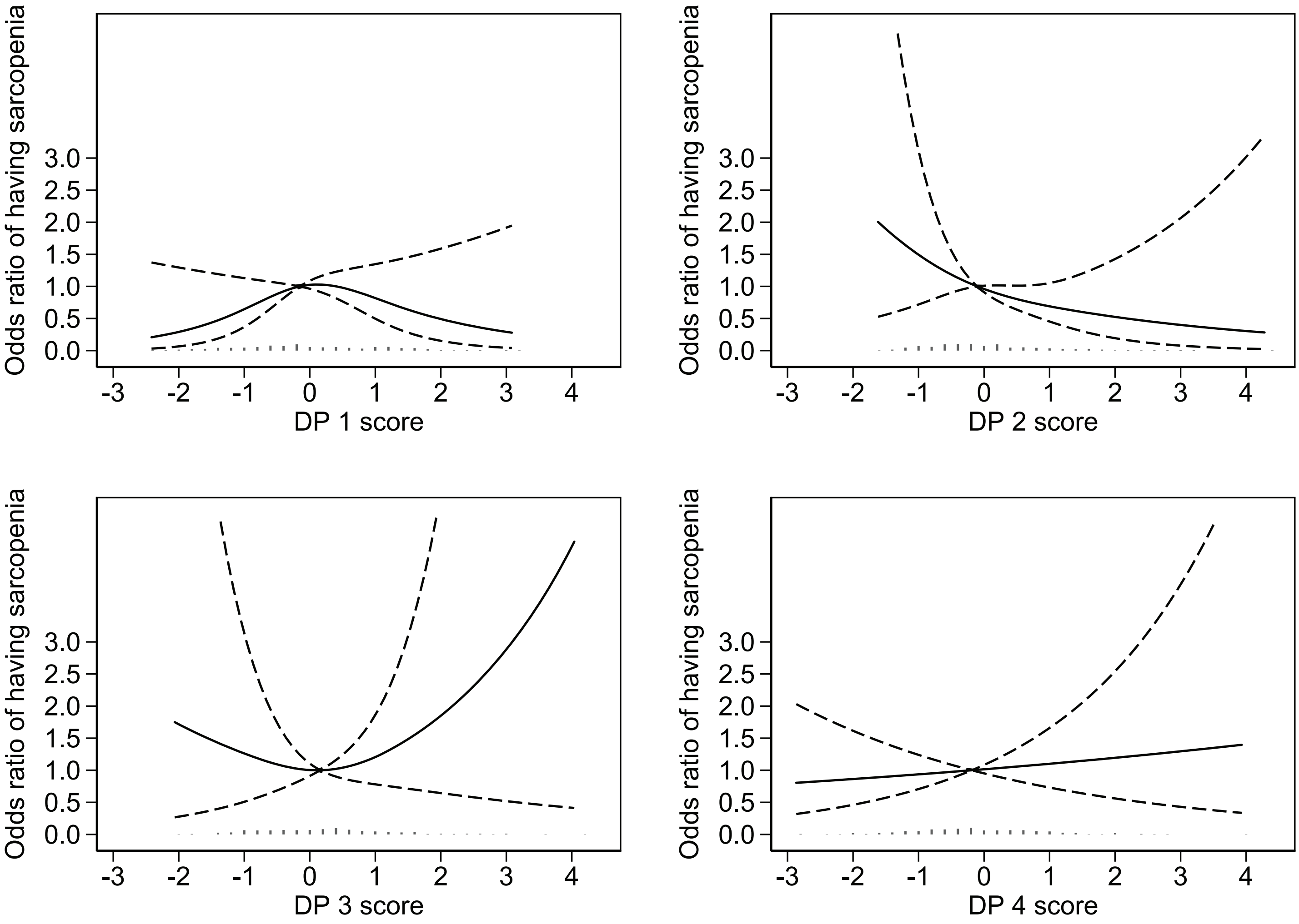

Supplementary Figure 3
Restricted cubic spline curves (3 knots placed at the 10th, 50th and 90th percentiles, with the median as reference point) of the associations between continuous increment (per standard deviation) in each dietary pattern (DP) at baseline, and the prevalence of sarcopenia at follow-up (n= 257).

Adjusted for age (continuous), reported energy intake (continuous), education (categorical), physical activity (categorical), smoking (categorical), comorbidity (categorical), body mass index (continuous), all at baseline, and follow-up period, i.e. Model 3.

The solid line represents the odds ratio and the dotted lines represents its 95% confidence interval. The spikes represent the distribution of adherence to the dietary pattern. The dotted vertical line represents the cut-off value between low and medium adherence and the solid vertical line represents the cut-off value between medium and high adherence to each DP. A score lower than the dotted vertical line corresponds to a low adherence to the current DP and a score higher than the vertical solid line corresponds to a high adherence to the current DP. A score between the dotted and the solid vertical line relates to a medium adherence to the current DP.

Supplementary Material 1

Pre-coded menu book used when collecting dietary intake during seven consecutive days at baseline.

We acknowledge Inga-Britt Gustafsson and Lars Berglund (Department of Public Health and Caring Sciences, Uppsala University) for the work they invested in the development of this menu book.

The menu book has been described and validated in

1. Rosell M, Hellénius M-L, de Faire U, Berglund L, Gustafsson I-B, Johansson G. (2003) Contribution of a manually coded part in an optically readable, precoded seven-day food record for the intake of energy, nutrients and foods. Scand J Nutr 47(3):123-131. https://doi.org/10.1080/11026480310014702

2. Nydahl M, Gustafsson IB, Mohsen R, Becker W. (2009) Comparison between optical readable and open-ended weighed food records. Food Nutr Res 53. https://doi.org/10.3402/fnr.v53i0.1889

UPPSALA UNIVERSITET
Institutionen för geriatrik

Inga anteckningar får göras i området utanför linjen!

MATDAGBOK

Frågehäfte

Namn: ....

Personnummer

	År		Mån		Dag		–	Nummer			
0	⬭	⬭	⬭	⬭	⬭	⬭	–	⬭	⬭	⬭	⬭
1	⬭	⬭	⬭	⬭	⬭	⬭	–	⬭	⬭	⬭	⬭
2	⬭	⬭		⬭	⬭	⬭	–	⬭	⬭	⬭	⬭
3	⬭	⬭		⬭	⬭	⬭	–	⬭	⬭	⬭	⬭
4	⬭	⬭		⬭		⬭	–	⬭	⬭	⬭	⬭
5	⬭	⬭		⬭		⬭	–	⬭	⬭	⬭	⬭
6	⬭	⬭		⬭		⬭	–	⬭	⬭	⬭	⬭
7	⬭	⬭		⬭		⬭	–	⬭	⬭	⬭	⬭
8	⬭	⬭		⬭		⬭	–	⬭	⬭	⬭	⬭
9	⬭	⬭		⬭		⬭	–	⬭	⬭	⬭	⬭

Dag

- ⬭ Måndag
- ⬭ Tisdag
- ⬭ Onsdag
- ⬭ Torsdag
- ⬭ Fredag
- ⬭ Lördag
- ⬭ Söndag

FRUKOST/MORGONMÅL

Plats	Tid (kl)		
Hemma	5:01 – 6:00	13:01 – 14:00	21:01 – 22:00
Mat hemifrån	6:01 – 7:00	14:01 – 15:00	22:01 – 23:00
Matsal arbete	7:01 – 8:00	15:01 – 16:00	23:01 – 24:00
Restaurang/Bar	8:01 – 9:00	16:01 – 17:00	24:01 – 1:00
Gatukök/Kiosk	9:01 – 10:00	17:01 – 18:00	1:01 – 2:00
Konditori/Kafé	10:01 – 11:00	18:01 – 19:00	2:01 – 3:00
Annan plats	11:01 – 12:00	19:01 – 20:00	3:01 – 4:00
	12:01 – 13:00	20:01 – 21:00	4:01 – 5:00

Vad åt jag till frukost och hur mycket?

Dryck	Antal ½ 1 2 3 4	
Kaffe		koppar
Te		koppar
Chokladdryck		koppar
Kaffegrädde i te/kaffe		matsked
Socker i dryck		bitar/tsk
Juice		dl
Nektar		glas
Saft		glas
Lättmjölk		glas
Mellanmjölk 1,5%		glas
Standardmjölk 3%		glas
Gårdsmjölk		glas

Fil, gröt m m	Antal ½ 1 2 3 4	
Lättfil, lättyoghurt		dl
Fil, naturell yoghurt, kefir, långfil, A-fil		dl
Fil, 1,5% fett		dl
Fruktyoghurt		dl
Fruktyoghurt		bägare
Gröt på havre, råg, graham, fullkornsvälling		dl
Gröt på manna, ris, annan välling		dl
Flingor, müsli		matsked
Sylt, sirap, honung		tesked
Extra socker		tesked

Bröd	Antal 1 2 3 4	
Fullkornsbröd eller mjukt grovt bröd		skivor
Vitt matbröd		skivor
Limpa		skivor
Hårt bröd, rågknäcke		bitar
Tunnbröd, veteknäcke		bitar

Pålägg	Antal 1 2 3 4	
Smör på		smörgåsar
Bregott på		smörgåsar
Hushållsmargarin på		smörgåsar
Bordsmargarin 80%* på		smörgåsar
Bordsmargarin 60%* på		smörgåsar
Lättmargarin 40%* på		smörgåsar
Ost, 17% fett på		smörgåsar
Ost, 28% fett på		smörgåsar
Ostskivor per smörgås		st
Kött, skinka på		smörgåsar
Korv på		smörgåsar
Leverpastej på		smörgåsar
Kaviar, rom på		smörgåsar
Marmelad, sylt, gelé på		smörgåsar

* 40%, 60%, 80% anger fetthalten på produkten

Övrigt	Antal ½ 1 2 3 4	
Ägg		st
Keso		matsked

Annat (beskriv vad och hur mycket)

Inga anteckningar får göras i området utanför linjen!

LUNCH/MITT PÅ DAGEN MÅL

Plats

- Hemma
- Mat hemifrån
- Matsal arbete
- Restaurang/Bar
- Gatukök/Kiosk
- Konditori/Kafé
- Annan plats

Tid (kl)

5:01 – 6:00	13:01 – 14:00	21:01 – 22:00
6:01 – 7:00	14:01 – 15:00	22:01 – 23:00
7:01 – 8:00	15:01 – 16:00	23:01 – 24:00
8:01 – 9:00	16:01 – 17:00	24:01 – 1:00
9:01 – 10:00	17:01 – 18:00	1:01 – 2:00
10:01 – 11:00	18:01 – 19:00	2:01 – 3:00
11:01 – 12:00	19:01 – 20:00	3:01 – 4:00
12:01 – 13:00	20:01 – 21:00	4:01 – 5:00

Vad åt jag till lunch/mitt på dagen mål samt hur mycket?

Kött, korv och inälvsmat	Portion, storlek ½ 1 2 3 4
Kött från nöt, kalv, lamm, vilt	
– Gryta	○ ○ ○ ○ ○
– Helt eller skivat kött	○ ○ ○ ○ ○
Griskött	
– Sidfläsk, bacon	○ ○ ○ ○ ○
– Gryta	○ ○ ○ ○ ○
– Helt eller skivat kött	○ ○ ○ ○ ○
Köttfärs	○ ○ ○ ○ ○
Korv	○ ○ ○ ○ ○
Lever	○ ○ ○ ○ ○
Blodpudding, blodkorv	○ ○ ○ ○ ○

Kyckling och annan fågel	Portion, storlek ½ 1 2 3 4
Gryta	○ ○ ○ ○ ○
Helt kött	○ ○ ○ ○ ○

Fisk och skaldjur	Portion, storlek ½ 1 2 3 4
Fisk, hel och filé	○ ○ ○ ○ ○
Fiskgryta	○ ○ ○ ○ ○
Fiskpinnar, -panetter	○ ○ ○ ○ ○
Fiskbullar, -gratäng	○ ○ ○ ○ ○
Gravad, inlagd, saltad eller rökt fisk	○ ○ ○ ○ ○
Skaldjur	○ ○ ○ ○ ○

Blandade sallader	Portion, storlek ½ 1 2 3 4
Med kött/kyckling	○ ○ ○ ○ ○
Med fisk/skaldjur	○ ○ ○ ○ ○
Med ost	○ ○ ○ ○ ○

Ris och pasta	Portion, storlek ½ 1 2 3 4
Ris	○ ○ ○ ○ ○
Makaroner, spagetti m m	○ ○ ○ ○ ○

Potatis	Antal ½ 1 2 3 4	
Kokt potatis (medelstor)	○ ○ ○ ○ ○	st
Bakad potatis	○ ○ ○ ○ ○	st

	Portion, storlek ½ 1 2 3 4
Potatismos, stuvad potatis	○ ○ ○ ○ ○
Stekt potatis	○ ○ ○ ○ ○
Pommes frites	○ ○ ○ ○ ○
Potatisgratäng	○ ○ ○ ○ ○
Potatissallad	○ ○ ○ ○ ○

Rotfrukter, grönsaker, bönor och svamp	Portion, storlek ½ 1 2 3 4
Morötter och andra rotfrukter	○ ○ ○ ○ ○
Bruna bönor	○ ○ ○ ○ ○
Ärtor, grönsaksblandning	○ ○ ○ ○ ○
Stuvade grönsaker	○ ○ ○ ○ ○
Blandad sallad	○ ○ ○ ○ ○
Vitkålssallad	○ ○ ○ ○ ○
Stekt lök	○ ○ ○ ○ ○
Blomkål, brysselkål, broccoli	○ ○ ○ ○ ○
Spenat	○ ○ ○ ○ ○
Andra grönsaker	○ ○ ○ ○ ○
Svamp	○ ○ ○ ○ ○

	Antal ½ 1 2 3 4	
Gurka	○ ○ ○ ○ ○	skivor
Tomat	○ ○ ○ ○ ○	st

forts...

Inga anteckningar får göras i området utanför linjen!

LUNCH/MITT PÅ DAGEN MÅL

Övriga rätter och tillbehör	Antal ½	1	2	3	4	
Pannkakor						st
Potatisbullar, palt kroppkakor						st
Pizza						st
Ärtsoppa						dl
Köttsoppa						dl
Fruktyoghurt						dl
Fruktyoghurt						bägare
Fil, yoghurt, kefir m m						dl
Gröt på havre, råg, graham, fullkornsvälling						dl
Gröt på manna, ris, annan välling						dl
Flingor, müsli						matsked
Ägg						st
Keso						matsked
Sylt, gelé						matsked
Senap, ketchup						matsked
Brun eller vit sås, sky						dl
Majonnäs, remoulad-bearnaisesås						matsked

Bröd	Antal 1	2	3	4	
Fullkornsbröd eller mjukt grovt bröd					skivor
Vitt matbröd					skivor
Limpa					skivor
Hårt bröd, rågknäcke					bitar
Tunnbröd, veteknäcke					bitar

Pålägg	Antal 1	2	3	4	
Smör på					smörgåsar
Bregott på					smörgåsar
Hushållsmargarin på					smörgåsar
Bordsmargarin 80%* på					smörgåsar
Bordsmargarin 60%* på					smörgåsar
Lättmargarin 40%* på					smörgåsar
Ost, 17% fett på					smörgåsar
Ost, 28% fett på					smörgåsar
Ostskivor per smörgås					st
Kött, skinka på					smörgåsar
Korv på					smörgåsar
Leverpastej på					smörgåsar
Kaviar, rom på					smörgåsar
Marmelad, sylt, gelé på					smörgåsar

* 40%, 60%, 80% anger fetthalten på produkten

Frukt och efterrätter	Antal ½	1	2	3	4	
Äpplen/päron						st
Apelsiner/mandariner samt grapefrukt						st
Bananer						st
Annan färsk frukt						st
Glasspinne, -strut						st
Glass						dl
Vispgrädde						matsked
Extra socker						tesked
Bär, färska eller frysta						dl
Konserverad frukt eller bär						dl

Dryck	Antal ½	1	2	3	4	
Kaffe						koppar
Te						koppar
Chokladdryck						koppar
Kaffegrädde i te/kaffe						msk
Socker i dryck						bitar/tsk
Juice						dl
Nektar						glas
Saft						glas
Läskedryck						glas
Lättmjölk						glas
Mellanmjölk 1,5%						glas
Standardmjölk 3%						glas
Gårdsmjölk						glas
Mineralvatten						glas
Vatten						glas
Lättöl						glas
Folköl						glas
Starköl						glas
Vin						glas
Sprit						cl

Annat (beskriv vad och hur mycket)

Inga anteckningar får göras i området utanför linjen!

MIDDAG/KVÄLLSMÅL

Plats	Tid (kl)		
Hemma	5:01 – 6:00	13:01 – 14:00	21:01 – 22:00
Mat hemifrån	6:01 – 7:00	14:01 – 15:00	22:01 – 23:00
Matsal arbete	7:01 – 8:00	15:01 – 16:00	23:01 – 24:00
Restaurang/Bar	8:01 – 9:00	16:01 – 17:00	24:01 – 1:00
Gatukök/Kiosk	9:01 – 10:00	17:01 – 18:00	1:01 – 2:00
Konditori/Kafé	10:01 – 11:00	18:01 – 19:00	2:01 – 3:00
Annan plats	11:01 – 12:00	19:01 – 20:00	3:01 – 4:00
	12:01 – 13:00	20:01 – 21:00	4:01 – 5:00

Vad åt jag till middag/kvällsmål samt hur mycket?

Kött, korv och inälvsmat
Portion, storlek: ½ 1 2 3 4

Kött från nöt, kalv, lamm, vilt
- – Gryta
- – Helt eller skivat kött

Griskött
- – Sidfläsk, bacon
- – Gryta
- – Helt eller skivat kött

Köttfärs
Korv
Lever
Blodpudding, blodkorv

Kyckling och annan fågel
Portion, storlek: ½ 1 2 3 4

Gryta
Helt kött

Fisk och skaldjur
Portion, storlek: ½ 1 2 3 4

Fisk, hel och filé
Fiskgryta
Fiskpinnar, -panetter
Fiskbullar, -gratäng
Gravad, inlagd, saltad eller rökt fisk
Skaldjur

Blandade sallader
Portion, storlek: ½ 1 2 3 4

Med kött/kyckling
Med fisk/skaldjur
Med ost

Ris och pasta
Portion, storlek: ½ 1 2 3 4

Ris
Makaroner, spagetti m m

Potatis
Antal: ½ 1 2 3 4

Kokt potatis (medelstor) st
Bakad potatis st

Portion, storlek: ½ 1 2 3 4

Potatismos, stuvad potatis
Stekt potatis
Pommes frites
Potatisgratäng
Potatissallad

Rotfrukter, grönsaker, bönor och svamp
Portion, storlek: ½ 1 2 3 4

Morötter och andra rotfrukter
Bruna bönor
Ärtor, grönsaksblandning
Stuvade grönsaker
Blandad sallad
Vitkålssallad
Stekt lök
Blomkål, brysselkål, broccoli
Spenat
Andra grönsaker
Svamp

Antal: ½ 1 2 3 4

Gurka skivor
Tomat st

forts...

Inga anteckningar får göras i området utanför linjen!

MIDDAG/KVÄLLSMÅL

Övriga rätter och tillbehör

Övriga rätter och tillbehör	½	1	2	3	4	
Pannkakor						st
Potatisbullar, palt kroppkakor						st
Pizza						st
Ärtsoppa						dl
Köttsoppa						dl
Fruktyoghurt						dl
Fruktyoghurt						bägare
Fil, yoghurt, kefir m m						dl
Gröt på havre, råg, graham, fullkornsvälling						dl
Gröt på manna, ris, annan välling						dl
Flingor, müsli						matsked
Ägg						st
Keso						matsked
Sylt, gelé						matsked
Senap, ketchup						matsked
Brun eller vit sås, sky						dl
Majonnäs, remouladbearnaisesås						matsked

Bröd

Bröd	1	2	3	4	
Fullkornsbröd eller mjukt grovt bröd					skivor
Vitt matbröd					skivor
Limpa					skivor
Hårt bröd, rågknäcke					bitar
Tunnbröd, veteknäcke					bitar

Pålägg

Pålägg	1	2	3	4	
Smör på					smörgåsar
Bregott på					smörgåsar
Hushållsmargarin på					smörgåsar
Bordsmargarin 80%* på					smörgåsar
Bordsmargarin 60%* på					smörgåsar
Lättmargarin 40%* på					smörgåsar
Ost, 17% fett på					smörgåsar
Ost, 28% fett på					smörgåsar
Ostskivor per smörgås					st
Kött, skinka på					smörgåsar
Korv på					smörgåsar
Leverpastej på					smörgåsar
Kaviar, rom på					smörgåsar
Marmelad, sylt, gelé på					smörgåsar

* 40%, 60%, 80% anger fetthalten på produkten

Frukt och efterrätter

Frukt och efterrätter	½	1	2	3	4	
Äpplen/päron						st
Apelsiner/mandariner samt grapefrukt						st
Bananer						st
Annan färsk frukt						st
Glasspinne, -strut						st
Glass						dl
Vispgrädde						matsked
Extra socker						tesked
Bär, färska eller frysta						dl
Konserverad frukt eller bär						dl

Dryck

Dryck	½	1	2	3	4	
Kaffe						koppar
Te						koppar
Chokladdryck						koppar
Kaffegrädde i te/kaffe						msk
Socker i dryck						bitar/tsk
Juice						dl
Nektar						glas
Saft						glas
Läskedryck						glas
Lättmjölk						glas
Mellanmjölk 1,5%						glas
Standardmjölk 3%						glas
Gårdsmjölk						glas
Mineralvatten						glas
Vatten						glas
Lättöl						glas
Folköl						glas
Starköl						glas
Vin						glas
Sprit						cl

Annat (beskriv vad och hur mycket)

Inga anteckningar får göras i området utanför linjen!

ÖVRIGT ÄTANDE

Notera dagens mellanmål och småmål – fika, frukt, godis etc

Om Du dricker kaffe/te/vatten vid olika tidpunkter ska Du summera respektive dryck. Har Du ätit mellanmål på flera platser, välj den vanligaste platsen.

Förmiddag kl 5 – 12<br>Plats	Livsmedel/Maträtt	Antal/Enhet<br>½ 1 2 3 4 5 6	
○ Hemma	Kaffe	○ ○ ○ ○ ○ ○ ○	koppar
○ Mat hemifrån	Te	○ ○ ○ ○ ○ ○ ○	koppar
○ Matsal arbete	Socker	○ ○ ○ ○ ○ ○ ○	bitar/tsk
○ Restaurang/Bar	Vatten	○ ○ ○ ○ ○ ○ ○	glas
○ Gatukök/Kiosk			
○ Konditori/Kafé	....	○ ○ ○ ○ ○ ○ ○	....
○ Annan plats			
	....	○ ○ ○ ○ ○ ○ ○	....
	....	○ ○ ○ ○ ○ ○ ○	....
	....	○ ○ ○ ○ ○ ○ ○	....

Eftermiddag kl 12 – 18<br>Plats	Livsmedel/Maträtt	Antal/Enhet<br>½ 1 2 3 4 5 6	
○ Hemma	Kaffe	○ ○ ○ ○ ○ ○ ○	koppar
○ Mat hemifrån	Te	○ ○ ○ ○ ○ ○ ○	koppar
○ Matsal arbete	Socker	○ ○ ○ ○ ○ ○ ○	bitar/tsk
○ Restaurang/Bar	Vatten	○ ○ ○ ○ ○ ○ ○	glas
○ Gatukök/Kiosk			
○ Konditori/Kafé	....	○ ○ ○ ○ ○ ○ ○	....
○ Annan plats			
	....	○ ○ ○ ○ ○ ○ ○	....
	....	○ ○ ○ ○ ○ ○ ○	....
	....	○ ○ ○ ○ ○ ○ ○	....

Kväll/natt kl 18 – 5<br>Plats	Livsmedel/Maträtt	Antal/Enhet<br>½ 1 2 3 4 5 6	
○ Hemma	Kaffe	○ ○ ○ ○ ○ ○ ○	koppar
○ Mat hemifrån	Te	○ ○ ○ ○ ○ ○ ○	koppar
○ Matsal arbete	Socker	○ ○ ○ ○ ○ ○ ○	bitar/tsk
○ Restaurang/Bar	Vatten	○ ○ ○ ○ ○ ○ ○	glas
○ Gatukök/Kiosk			
○ Konditori/Kafé	....	○ ○ ○ ○ ○ ○ ○	....
○ Annan plats			
	....	○ ○ ○ ○ ○ ○ ○	....
	....	○ ○ ○ ○ ○ ○ ○	....
	....	○ ○ ○ ○ ○ ○ ○	....

Inga anteckningar får göras i området utanför linjen!

Supplementary Material 2

A common concern in studies involving food intake is underreporting of dietary intake and thus an increased risk of misclassification. In our main analysis we excluded extreme outliers and adjusted for reported total energy intake. This allows ranking of individuals' dietary intakes, which is a most important aspect when studying associations, and is one approach when dealing with risk of misclassification[1; 2]. If, on the other hand, the interest is in the actual dietary intake to make quantitative assessments, it may be more important to have adequate reporters. A commonly used method to identify adequate energy reporters is the Goldberg cut-offs[3].

The application of the Goldberg cut-offs is based on total energy requirements and are therefore dependent on information on the basal metabolic rate (BMR) and the physical activity level (PAL). We used the Harris Benedict equation[4] for calculation of BMR, as it has been reported to be fairly valid compared to indirect calorimetry in this study population, however at the age of 82 years[5]. In the present study, self-reported information on physical activity is based on four questions that do not in detail specify the intensity or amount of activity performed. The leisure-time physical activity levels were defined as sedentary, moderate, regular or athletic and translated into PAL 1.4, 1.5, 1.6, and 1.7, respectively. Individual PALs were used when defining the Goldberg cut-offs.

In this sensitivity analysis, 44 out of 1133 men included in the main analysis were excluded due to missing information on BMR or PAL, and 855 (out of 1089) men were defined as adequate energy reporters according to the Goldberg cut-offs (229 were classified as under-reporters and 5 as over-reporters). There were some differences in baseline characteristics between the main study population and the subgroup of adequate energy reporters. The mean energy intake was higher in the subgroup of 216 adequate energy reporters (1954 kcal) compared to the 257 men in the main analysis (1853 kcal), see Supplementary Table G1.

As in our main analysis, dietary patterns (DPs) were again defined using principal component analysis, now in the subgroup of the 855 adequate reporters. The result of Bartlett's test of sphericity (<.001), and Kaiser-Meyer-Olkin measure of sampling (0.537), indicated that the factor analysis is useful with our data. The top four principal components were selected based on a combined assessment of a break point (elbow) in the scree plot and eigenvalues (>1.5). The identified principal components were rotated with varimax and the chosen DPs accounted for 26.9 % of the total variance. The derived DPs were equivalent to the DPs derived in the main analysis, however with some minor differences in loadings (Supplementary Table G2).

The association between each DP and the prevalence of sarcopenia was analyzed using logistic regression models estimating odds ratios (ORs) adjusted according to Model 3 (age at baseline, follow-up period, reported energy intake at baseline, education, physical activity at baseline, smoking, morbidity at baseline and BMI at baseline). The results for DP2 were largely in line with those in the main analyses, however with wider confidence intervals (Supplementary Table G3), potentially attributed to a smaller population size.

However, both BMR and physical activity is, in their self, prone to misclassification. This means that by trying to overcome one type of bias (underreporting), we might introduce other types of bias. Furthermore, using the Goldberg method does not distinguish between the actual dietary intake and the reported intake, it detects low levels of reported energy intake.

References

1. Willett W (2013) Nutritional epidemiology, 3rd ed., vol. 40, pp. 206-286. Oxford: Oxford University Press.
2. Willett W (2013) Nutritional epidemiology, 3rd ed., vol. 40, pp. 305-333. Oxford: Oxford University Press.
3. Black AE (2000) Critical evaluation of energy intake using the Goldberg cut-off for energy intake:basal metabolic rate. A practical guide to its calculation, use and limitations. Int J Obes Relat Metab Disord 24, 1119-1130.
4. Harris JA, Benedict FG (1918) A Biometric Study of Human Basal Metabolism. Proc Natl Acad Sci U S A 4, 370-373.
5. Karlsson M, Olsson E, Becker W et al. (2017) Ability to predict resting energy expenditure with six equations compared to indirect calorimetry in octogenarian men. Exp Gerontol 92, 52-55.

Supplementary Table G1

Characteristics at baseline (mean age 71) and follow-up (mean age 87) of men included in the subgroup analysis (n=216) and grouped according to low respectively high adherence to each dietary pattern at baseline.

		Total	DP 1		DP 2		DP 3		DP 4	
			Low	High	Low	High	Low	High	Low	High
		n=216	n=79	n=76	n=67	n=81	n=63	n=74	n=92	n=59
Age (years)	baseline	70.9 (0.6)	70.9 (0.6)	71.0 (0.6)	70.9 (0.6)	70.9 (0.6)	70.8 (0.6)	70.9 (0.6)	70.9 (0.5)	70.9 (0.6)
Follow-up period (years)		15.6 (0.7)	15.6 (0.7)	15.6 (0.7)	15.7 (0.7)	15.5 (0.7)	15.5 (0.7)	15.6 (0.6)	15.6 (0.7)	15.6 (0.6)
Body weight (kg)	baseline	78.3 (9.4)	78.5 (8.6)	77.2 (10.3)	79.6 (10.0)	78.4 (8.7)	78.2 (10.2)	77.7 (8.3)	78.1 (9.9)	78.5 (9.2)
Body weight (kg)	follow-up	75.3 (11.3)	74.7 (9.7)	74.5 (13.1)	76.0 (13.6)	76.4 (9.9)	73.5 (11.6)	75.4 (9.8)	75.5 (12.4)	75.2 (11.0)
BMI (kg/m^2)	baseline	25.6 (2.8)	25.4 (2.6)	25.4 (3.0)	26.2 (3.1)	25.3 (2.6)	25.6 (3.1)	25.2 (2.4)	25.5 (2.9)	25.6 (2.9)
BMI (kg/m^2)	follow-up	25.4 (3.5)	25.0 (2.9)	25.2 (4.1)	25.8 (4.5)	25.4 (3.0)	24.8 (3.5)	25.2 (3.0)	25.4 (3.8)	25.3 (3.5)
Appendicular LMI (kg/m^2)	follow-up	7.5 (0.8)	7.4 (0.8)	7.5 (0.9)	7.5 (0.9)	7.5 (0.7)	7.4 (0.8)	7.5 (0.7)	7.5 (0.8)	7.4 (0.7)
Reported energy intake (kcal/day)	baseline	1954 (386)	1834 (307)	2085 (424)	1860 (361)	2031 (410)	1742 (302)	2145 (417)	1968 (364)	2012 (415)
Reported protein intake (g/day)	baseline	72 (15)	67 (12)	76 (17)	68 (13)	76 (17)	63 (10)	80 (16)	70 (13)	77 (17)
Reported protein intake (g/kg body weight/day)	baseline	0.93 (0.23)	0.86 (0.18)	1.01 (0.27)	0.86 (0.20)	0.98 (0.23)	0.82 (0.15)	1.03 (0.23)	0.90 (0.20)	1.00 (0.27)
Years of education (%)										
6-7 years		48%	29%	61%	51%	40%	44%	50%	41%	56%
8-13 years		33%	39%	28%	36%	30%	32%	26%	34%	32%
> 13 years		19%	32%	12%	13%	31%	24%	24%	25%	12%
Physical activity level (%)	baseline									
Low		31%	33%	33%	28%	30%	37%	27%	32%	24%
Medium		61%	57%	66%	67%	60%	52%	64%	58%	73%
High		8%	10%	1%	4%	10%	11%	9%	11%	3%
Smoking (%)	baseline									
Never		43%	44%	45%	42%	43%	43%	39%	48%	36%
Current		8%	8%	9%	12%	5%	13%	5%	7%	8%
Former		49%	48%	46%	46%	52%	44%	55%	46%	56%
Charlson Comorbidity Index (%)	baseline									
0		80%	78%	79%	78%	80%	78%	82%	77%	90%
≥1		20%	22%	21%	22%	20%	22%	18%	23%	10%

Values are presented as mean (standard deviation) for continuous measures, and as percentage for categorical measures.
BMI, body mass index; LMI, lean mass index

Supplementary Table G2

Dietary patterns and principal components loadings for each of the food group. Derived in a subgroup (n=855) with adequate reporters (defined using Goldberg's cut-off)

**Food groups**[a]	**DP1**	**DP2**	**DP3**	**DP4**
Bread	-0.0128	0.0328	**0.6101**	0.0744
Cheese	-0.0310	-0.0588	**0.5550**	-0.0894
Fermented milk	-0.1728	0.0220	0.1967	**-0.4043**
Milk	**0.4449**	0.0317	-0.0301	0.1879
Soup	0.0064	0.1709	0.0115	-0.1903
Pancake	0.2493	-0.0678	-0.0558	-0.1068
Quiche/pie	-0.0327	-0.0621	-0.0758	-0.0587
Salad with protein	-0.2655	0.1510	-0.0077	0.0786
Cold meats	-0.0640	0.0873	0.1698	0.1660
Meat	0.0579	-0.0250	0.1457	**0.4500**
Poultry	-0.0931	**0.3329**	-0.0855	0.0841
Egg	-0.0920	0.0744	-0.0728	**0.3438**
Seafood	-0.0606	0.0870	-0.0828	0.0124
Vegetables	0.0746	**0.5066**	0.0174	-0.0004
Green salad	-0.1248	**0.3074**	0.1383	0.0532
Legumes	0.1458	-0.0124	-0.0135	0.1028
Juice	-0.0673	0.1944	-0.0185	-0.2343
Fruit	0.0729	**0.4704**	0.0266	-0.0936
Pasta and rice	0.0126	**0.4035**	-0.1028	0.0590
Potato	0.1137	0.0546	0.2879	**0.3023**
Cereals	**0.4778**	0.0780	-0.0801	0.1085
Desserts and pastry	0.1549	0.0214	-0.0396	**-0.3173**
Marmalade, jam and sugar	0.1183	-0.0151	0.2452	-0.1710
Soft drink	0.0928	0.0821	0.0802	-0.1201
Alcohol <4 %	**-0.3922**	-0.0907	-0.0571	0.1987
Alcohol >4 %	**-0.3392**	0.0585	-0.0984	0.0870
Proportion of variability explained (%)[b]	7.78	6.97	6.09	6.05

[a]Food groups are defined and exemplified in Supplementary Table 1
[b]Total variance explained by these four derived components equals 26.9%.
Principal components loadings >.30 and <-.30 in bold

Supplementary Table G3

Logistic regression analysis between adherence to each dietary pattern at baseline (mean age 71) and prevalence of sarcopenia defined according to EWGSOP2 at follow-up (mean age 87) in individuals in a subgroup with adequate reporters (defined using Goldberg's cut-off).

	Adherence to dietary pattern			Continuous
	**Low**	**Medium**	**High**	(per 1 SD increment)
**DP 1**				
Participants, n	79	61	76	216
Sarcopenia, n (%)	14 (18)	14 (23)	16 (21)	44 (20)
OR (95% CI), model 3[a]	1.00 (ref)	1.67 (0.64 - 4.32)	1.25 (0.49 - 3.18)	1.17 (0.81 - 1.70)
**DP 2**				
Participants, n	67	68	81	216
Sarcopenia, n (%)	15 (22)	15 (22)	14 (17)	44 (20)
OR (95% CI), model 3[a]	1.00 (ref)	0.81 (0.33 - 1.99)	0.61 (0.24 - 1.51)	0.70 (0.46 - 1.05)
**DP 3**				
Participants, n	63	79	74	216
Sarcopenia, n (%)	13 (21)	17 (22)	14 (19)	44 (20)
OR (95% CI), model 3[a]	1.00 (ref)	1.40 (0.54 - 3.62)	1.09 (0.40 - 3.02)	1.21 (0.82 – 1.78)
**DP 4**				
Participants, n	92	65	59	216
Sarcopenia, n (%)	17 (18)	14 (22)	13 (22)	44 (20)
OR (95% CI), model 3[a]	1.00 (ref)	1.31 (0.55 - 3.12)	1.36 (0.55 - 3.38)	1.03 (0.71 – 1.49)

DP, dietary pattern; OR, odds ratio; CI, confidence interval; SD, standard deviation.

Participants were categorized as low, medium or high adherent to each DP based on factor scores and according to tertiles limits determined based on adherence to dietary patterns at baseline, in a subgroup of 855 men, defined as adequate energy reporters according to Goldberg's cut-off.

[a] Model 3: adjusted for age at baseline (continuous), follow-up period (continuous), reported energy intake at baseline (continuous), education (categorical), physical activity at baseline (categorical), smoking (categorical), morbidity at baseline (categorical) and BMI at baseline (continuous).
